# Emerging roles for ABC transporters as virulence factors in uropathogenic *Escherichia coli*

**DOI:** 10.1073/pnas.2310693121

**Published:** 2024-04-12

**Authors:** Allyson E. Shea, Valerie S. Forsyth, Jolie A. Stocki, Taylor J. Mitchell, Arwen E. Frick-Cheng, Sara N. Smith, Sicily L. Hardy, Harry L. T. Mobley

**Affiliations:** ^a^Department of Microbiology and Immunology, University of Michigan Medical School, Ann Arbor, MI 48109; ^b^Department of Microbiology and Immunology, College of Medicine, University of South Alabama, Mobile, AL 36688

**Keywords:** transport, UTI, pathogenesis, UPEC

## Abstract

Urinary tract infections (UTI) account for a substantial financial burden globally. Over 75% of UTIs are caused by uropathogenic *Escherichia coli* (UPEC), which have demonstrated an extraordinarily rapid growth rate in vivo. This rapid growth rate appears paradoxical given that urine and the human urinary tract are relatively nutrient-restricted. Thus, we lack a fundamental understanding of how uropathogens propel growth in the host to fuel pathogenesis. Here, we used large in silico, in vivo, and in vitro screens to better understand the role of UPEC transport mechanisms and their contributions to uropathogenesis. In silico analysis of annotated transport systems indicated that the ATP-binding cassette (ABC) family of transporters was most conserved among uropathogenic bacterial species, suggesting their importance. Consistent with in silico predictions, we determined that the ABC family contributed significantly to fitness and virulence in the urinary tract: these were overrepresented as fitness factors in vivo (37.2%), liquid media (52.3%), and organ agar (66.2%). We characterized 12 transport systems that were most frequently defective in screening experiments by generating in-frame deletions. These mutant constructs were tested in urovirulence phenotypic assays and produced differences in motility and growth rate. However, deletion of multiple transport systems was required to achieve substantial fitness defects in the cochallenge murine model. This is likely due to genetic compensation among transport systems, highlighting the centrality of ABC transporters in these organisms. Therefore, these nutrient uptake systems play a concerted, critical role in pathogenesis and are broadly applicable candidate targets for therapeutic intervention.

## Significance

Transport is a vital process for survival across all domains of life. Employing multiple unbiased techniques, we show that the disruption of ATP-binding cassette (ABC) transporters leads to decreased fitness in uropathogenic *Escherichia coli*, the main causative agent of urinary tract infection. ABC transporters provide nutrients to the bacterial cell, requiring ATP. Loss of these systems leads to host-specific nutritional deficiencies that result in a dysregulation of virulence traits such as growth and motility. This highlights the overarching influence of bacterial transport and pleiotropic effects beyond bacterial metabolism. Deletion of multiple, specific, transcriptionally redundant systems leads to a decrease in murine colonization. Together, these findings extend our current knowledge of the cross-talk between bacterial metabolism and uropathogenic virulence mechanisms.

Fifty percent of women worldwide experience a urinary tract infection (UTI) at least once during their lives (1–3). UTIs are responsible for ~$5 billion a year in associated healthcare costs in the United States, making it the highest financial burden in kidney and urologic diseases (4–6). Of women visiting clinicians for a UTI, 95% do so for symptoms of cystitis (7). However, UTI range in severity because bacteria can ascend the ureters and cause acute pyelonephritis, elevating the risk of bacteremia, urosepsis, and fatality. Current treatment regimens of antibiotics are becoming increasingly ineffective due to rising resistance, it is therefore vital that we identify pathogen-specific therapies that target bacterial virulence (3, 8, 9). To understand bacterial genes important for infection, we constructed an ordered transposon library containing 2,419 unique gene mutants in prototype UPEC strain CFT073. This library was previously screened during experimental UTI to identify mutants that have significantly decreased fitness in the murine bladder (10). From 203 preliminary hits, we identified 20 clusters of orthologous genes and functional groups that are important for UPEC fitness during UTI. One such group was transporters, which composed nearly 25% of the identified fitness factors (10). These data suggest nutrient acquisition is a key determinant of UPEC urovirulence, thus prompting our independent, rigorous investigation into transport systems.

UPEC adherence, toxin activity, motility, and iron acquisition are well-studied virulence mechanisms (8, 11, 12), while bacterial energy acquisition strategies and metabolic flexibility have been historically understudied with respect to their role in virulence. However, our recent studies using RNA-seq on *Escherichia coli* in a UTI mouse model (13), and from human UTI (14), indicated that specific nutrient transporters are vital to UPEC within the urinary tract. While general bacterial biosynthetic pathways are repressed, import systems are induced (14) suggesting UPEC adopt a scavenging lifestyle during UTI relying on the consumption of host metabolites. We also know that the TCA cycle and gluconeogenesis, processes fueled by amino acids, are critical metabolic pathways utilized by UPEC during infection (15, 16). Collectively, these data from multifaceted studies provide strong evidence for the essential role of host-derived nutrients in fueling uropathogenesis.

There are six major transporter families annotated in UPEC including ATP-binding cassette (ABC) eukaryotic-like, ABC prokaryotic-like, phosphotransferase (PTS), major facilitator superfamily (MFS), solute carrier (SLC), and “Others” [KEGG, (17)]. We found that ABC prokaryotic-like uptake systems were the most critical for fitness in the ascending murine model of UTI using Tn-seq screening. The ABC eukaryotic-like transport systems in bacteria mainly act as efflux pumps to facilitate multidrug resistance (18–20) whereas the prokaryotic-like systems consist of a substrate-binding protein, permease, and an ATPase subunit (18). In addition to coupling ATP hydrolysis to transport substrates from the periplasm into the cytoplasm (21, 22), ABC systems can also regulate physiological processes both directly and indirectly (22–24). Unlike the other families of transporters, ABC prokaryotic-like transporters are generally encoded as polycistronic systems (24) and can contain multiple subunits that are able to form hetero- or homodimers (22, 23, 25). Due to the sequence variances between human and bacterial ABC systems, these could be potential targets for competitive pharmacological inhibition (25). Despite the wealth of literature on this family of transporters that spans the biological kingdoms, there are still several annotated but uncharacterized systems with unknown substrates.

We conducted an unbiased survey of all annotated transport systems of the type uropathogenic strain *E. coli* CFT073 (26, 27) to identify the most critical for the host-specific nutritional niche and infection-specific virulence in the host. We narrowed our focus to the family of ABC prokaryotic-like transporters, which were found to be highly conserved among both UPEC and other uropathogenic Gram-negative species. Deletion of ATP-driven transport systems caused severe growth defects and altered urovirulence phenotypes, such as motility. Finally, we determined that genetic redundancy of these transporters would require multisystem inhibition to significantly impede bacterial colonization of the urinary tract. For example, we found a quadruple mutant in highly redundant amino acid import systems necessary to achieve substantial fitness defects in vivo. Another critical advancement from this study was establishing a transporter hierarchy of which systems are favored during active UTI. Indeed, we found transporter hierarchy at the genetic level that showed primary upregulation of ABC systems. Together, these results posit that nutrient availability in the host niche is an important factor directly contributing to UPEC uropathogenic potential.

## Results

### Diverse Uropathogens Display High Conservation of ABC Transporters.

**Fig. 1.**
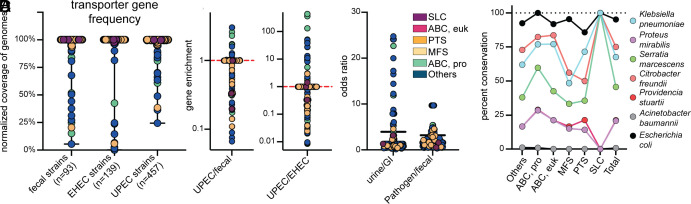
Diverse uropathogens display high conservation of ABC transporters. (*A*) All 640 KEGG-annotated transporter protein products in UPEC strains CFT073 were surveyed for homology against fecal (n = 93), enterohemorrhagic (n = 139), and uropathogenic (n = 457) isolates of *E. coli*, with a protein identity and coverage over 90% defining a hit. Each dot represents a single protein from strain CFT073 and indicates the relative number of strains it was detected in within each cohort, using an amino acid cutoff of >90%. These are color-coded based on the family of transporters: solute carrier (purple), ABC eukaryotic-like (pink), phosphotransferase (orange), major facilitator superfamily (yellow), ABC prokaryotic-like (green), and Others (blue). (*B*) An enrichment analysis was performed comparing each transporter in UPEC relative to either fecal or EHEC isolates. A value greater than 1 indicates an enrichment in UPEC genomes, and <1 indicates enrichment in fecal or EHEC genomes. The dashed red line indicated the median of these data. (*C*) Based on the prevalence determined, an odds ratio was calculated for each transport protein. The odds ratios in strains isolated from human urine compared to the gastrointestinal tract (GI, both fecal and EHEC) and pathogenic isolates (UPEC and EHEC) compared to commensal fecal isolates. (*D*) All *E. coli* CFT073 KEGG-annotated transporters were surveyed against the pathogenic type strains (uropathogenic or bacteremic) of six different bacterial species, in addition to the commonly used UPEC isolate UTI89. Dots indicate the percent conservation of each transport system in every species, displayed by family.

There are 640 KEGG-annotated transport loci in uropathogenic *E. coli* (UPEC) strain CFT073 that fall into six different families (17). To expand our understanding of the prevalence of these systems, we collated three large genome banks on the PATRIC database (28, 29) for UPEC (n = 457), enterohemorrhagic *E. coli* (EHEC) (n = 139), and fecal-isolated (n = 93) *E. coli* strains (Dataset S1). We identified the CFT073 transporters in these three cohorts using BLASTP with a >90% amino acid sequence identity cut-off (Fig. 1*A*). None of the CFT073 transporters are unique to CFT073, all of them were found in >24% of UPEC strains and >5% of fecal isolates. For each of the transporters, we were able to determine gene enrichment based on the frequency among UPEC compared to fecal isolate populations, and UPEC compared to EHEC isolates (Fig. 1*B*). The majority of transporters having a high enrichment, indicating increased prevalence In UPEC strains over the commensal fecal or EHEC strains, were from the Others and ABC prokaryotic transporter families. When examining transporter enrichment among UPEC compared to EHEC, systems for transport of capsular polysaccharides and iron–siderophore complexes were increased. When comparing strains of urine origin to gastrointestinal origin (both EHEC and commensal), we again saw the Others family exhibiting higher odds ratios with many systems of hypothetical or unknown function (Fig. 1*C*). In contrast to these comparisons, there were much lower odds ratios determined when comparing the two *E. coli* pathogens to commensal fecal isolates (Fig. 1*C*). Interestingly, half of the highest ratios in this group were in systems related to cytochrome C and heme, recently explored by other works (30).

Although >80% of UTI are caused by UPEC, other Gram-negative species are becoming increasingly more common (31, 32). The rate of non-UPEC and multispecies infections drastically increases when considering complicated UTI cases, such as those with catheterized or immunocompromised patients (31, 33). We proceeded to examine the amino acid sequences of all CFT073 transporters against notable Gram-negative urinary pathogens *Klebsiella pneumoniae*, *Proteus mirabilis*, *Serratia marcescens*, *Citrobacter freundii*, *Providencia stuartii*, *Acinetobacter baumannii*, in addition to *E. coli* cystitis strain UTI89 as a control (34) (*SI Appendix*, Fig. S1). The SLC family was unique in that all three systems were either completely present or absent in the pathogen. On average, each transporter was detected in 3.3/7 species. Overall, the ABC prokaryotic family had the largest percent conservation (53.9%) across species (Fig. 1*D*). In fact, GltL (the ATP-binding protein subunit of the ABC transporter for glutamate and aspartate import) was the only encoded transporter with >70% homology found in all 7 species (17, 35). At a transporter level, Glt, Opp, and Dpp were among the most conserved systems. From a therapeutic standpoint, this makes ABC importers an attractive potential candidate to target across diverse UTI-causing pathogens.

### Transport Systems Largely Reside in the Conserved Genome.

Pathogenic strains of *E. coli* have acquired genetic elements that contribute to their virulence which are often carried within pathogenicity islands (PAI). The genome of uropathogenic *E. coli* strain CFT073 has 13 annotated PAIs that represent 12.8% of the genome (36), which are the most genetically diverse regions. The remainder of the CFT073 genome is more highly conserved among the species; however, only 52.4% of this genomic content was found to be common among all pathotypes of *E. coli*, designated as the core genome (36). Roughly 92% of all transporters in the CFT073 genome reside within the conserved portion (*SI Appendix*, Fig. S2*A*). In many cases, there are regions in the genome that lack transport-related genes, overlapping with PAIs (gray) (*SI Appendix*, Fig. S2*A*). There are 640 KEGG-annotated transporters that fall into six different families. In addition, 466 loci (72.7%) and 81.2% of transport operons were available as transposon mutants in our previously constructed and validated *E. coli* CFT073 ordered library (10), which had a 53% genome saturation rate. The number of transporter genes in each of these families ranges from three (SLC) to 276 (Others) (*SI Appendix*, Fig. S2*B*) (17). All families had >60% representation in our library, and thus, all our experimental screens. We chose only a single insertional mutant, selected from our transposon library, to represent each transport gene. Mutants were curated to select for transposon insertion with the best proximity to the predicted translational start site (*SI Appendix*, Fig. S2*C*). By selecting these mutants, we increased the likelihood that mutations would result in a defective transport system.

### In Vivo Screen Revealed Organ Site Differences in the Hierarchy of Preferred Transporters.

Our previous study examined the gene expression of UPEC in the urine of female patients with uncomplicated UTI compared to culture in human urine in vitro (14) (Dataset S2). In general, transporter gene expression during urine growth in vitro correlated with patient samples (R^2^ = 0.538) (*SI Appendix*, Fig. S3*A*). However, select transporters were outliers in this dataset and among some of the most differentially regulated genes when compared to growth in human urine in vitro (*SI Appendix*, Fig. S3*B*). Many of the patient up-regulated transport systems were included in our transposon pooling: *ptsG*, *fruB*, and *yhfC*, among others. While we anticipated overlap with RNA-seq studies (13, 14), we found that gene upregulation was not indicative of fitness in the murine urine in vivo. In fact, a correlation model of gene expression and fitness factor was nonexistent (R^2^ = 0.002) (*SI Appendix*, Fig. S3*C*). This highlights the importance of using a Tn-seq approach, which yields different and additional findings of genetic interactions and quantitative fitness of single genes. The differences observed are likely due to the post-translational regulation and kinetics of genes and gene products, particularly relevant when examining transport systems.

**Fig. 2.**
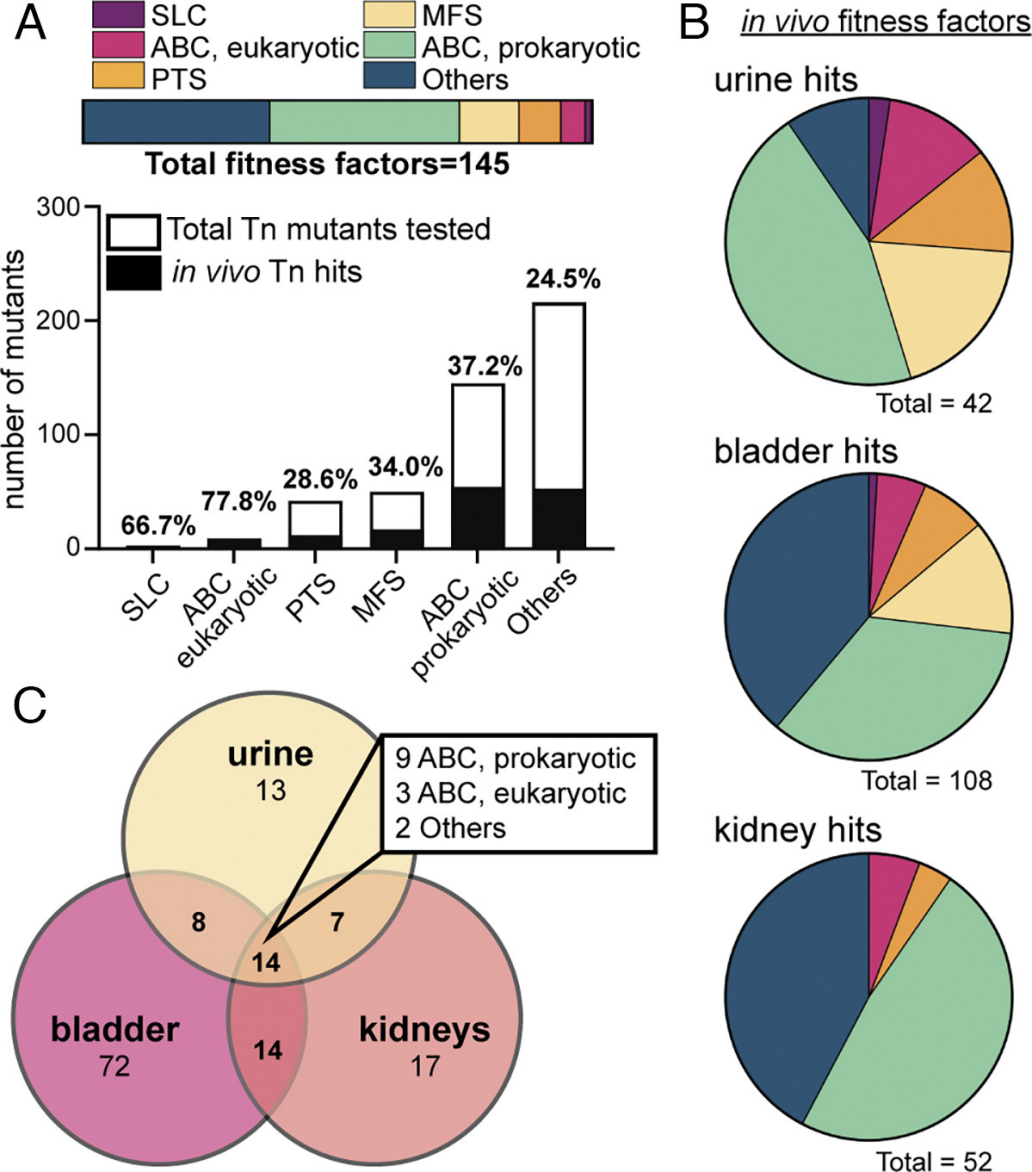
Assessing the fitness of transporter mutants in vivo using the murine model of ascending UTI. (*A*) The genes that are fitness factors in vivo (hits, black bar height) are displayed as a fraction of the total number of transposon mutants used in the experiment (total bar height); exact percentages are indicated above the bar. Transposon mutants were identified as hits in one or more organ sites if *P*_adj_ < 0.05. In addition, 145 unique mutants were classified as a hit; their assigned families (as a percentage of the total) are represented in the horizontal bar. (*B*) Each pie chart defines which transporter family each fitness factor is a member of in the indicated organ site. These are color-coded based on the family of transporters: solute carrier (purple), ABC eukaryotic-like (pink), phosphotransferase (orange), major facilitator superfamily (yellow), ABC prokaryotic-like (green), and Others (blue). The total number of mutants displaying fitness defects in that organ site is specified under the chart. (*C*) The Venn Diagram represents the total number of hits determined from murine urine (yellow), bladder (pink), and kidneys (orange). Many genes were detected as fitness factors in multiple organ sites, and 14 were detected in the urine, bladder, and kidney; the families of these 14 hits are indicated in the box.

We were unique in our Tn-seq experimental design by barcoding samples from each mouse to allow for independent analysis of individual animals in the cohort. This is in response to the challenging bottleneck during the ascending model of UTI that has been previously studied in detail (10, 37). To minimize bias in mutant output quantification introduced by stochastic loss or clonal expansion, we divided the available mutants into three pools: 1) the ABC families (n = 154), 2) the PTS, MFS, and SLC families (n = 95), and 3) the Others (n = 217) (*SI Appendix*, Fig. S4). Using Principal Component Analysis, we saw mouse organ samples (urine, bladder, and kidneys) that were outliers suggesting a bottleneck effect that have been previously described in the murine model of UTI (10, 37); interestingly, these did not correlate to the samples with the lowest CFU burden (*SI Appendix*, Fig. S4 *A*–*C*). When examining the fitness factors detected in at least one of three mouse collection sites, we noticed higher representation of the smaller transporter families such as SLC (66.7%) and ABC eukaryotic (77.8%) (Fig. 2*A* and Dataset S3). However, the total number of hits (n = 145) was largely those in the ABC prokaryotic-like and Others families (Fig. 2*A*). We noted that the profiles of fitness factors determined in each organ site were unique. The urine had mutants representing all six transport families, while the kidneys only had representation from four families (Fig. 2*B*). In both organs, the bladder and the kidneys, the ABC prokaryotic and Others were consistently the most represented families (Fig. 2*B*). When probing the common fitness factors detected among all three collection sites, the majority (9/14) were in the ABC prokaryotic family and collectively the ABC families made up ~86% of the common fitness factors (Fig. 2*C*). This was our first experimental evidence to suggest an important role for this particular class of transporters along the length of the heterogenous urinary tract.

### Culture In Vitro Yields Only Minimal Growth Defects and Deficiencies.

**Fig. 3.**
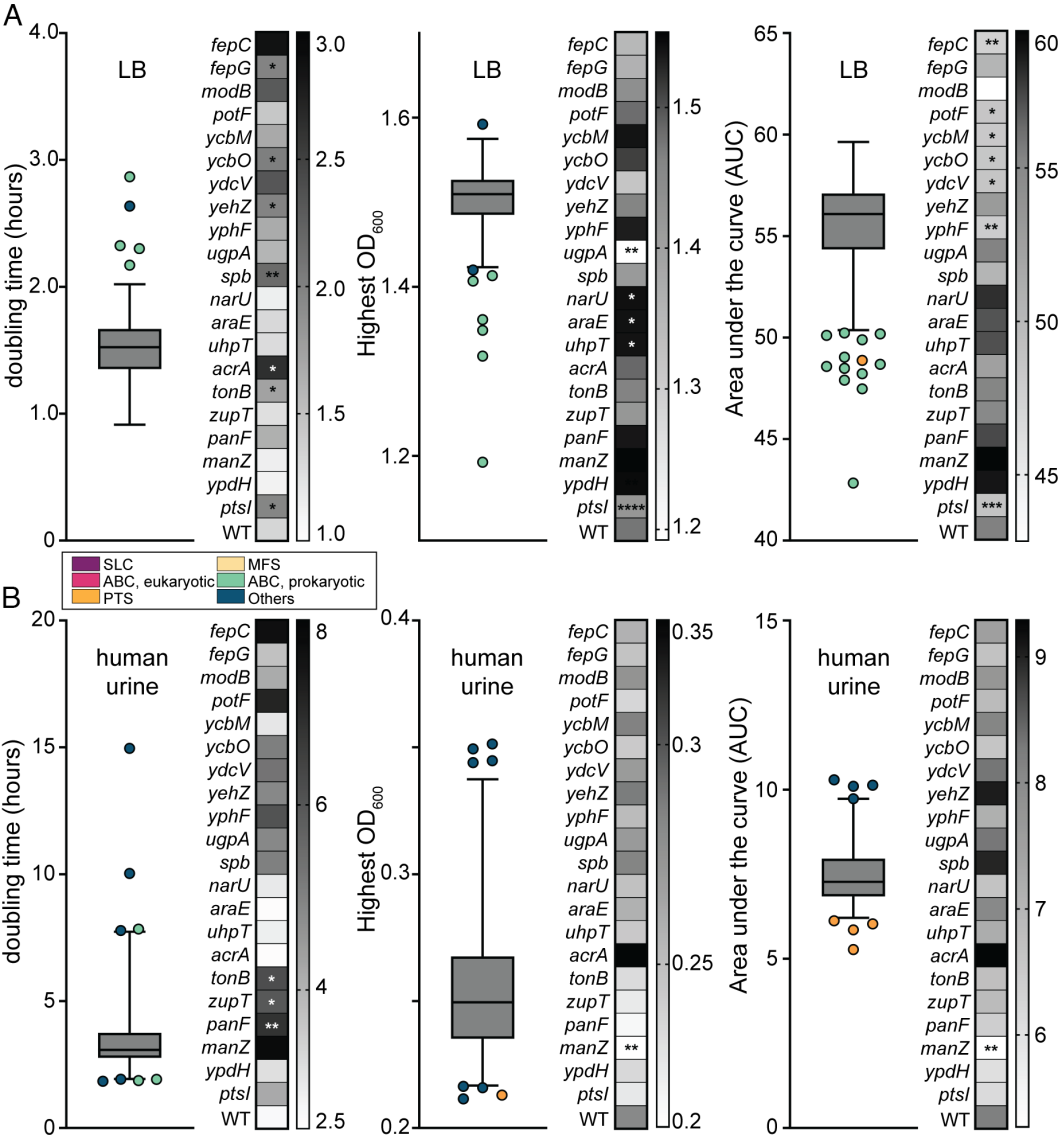
Growth defects of transporter mutants in LB and human urine in vitro. All 466 transposon mutants were cultured in (*A*) LB or (*B*) human urine (HU), alongside a wild-type control (n = 5). Three metrics were assessed to determine statistical differences in growth trends: doubling time (DT) in hours, saturating OD_600_, and area under the growth curve (AUC). The data from these screens are displayed as a box and whisker plot, indicating the median and outliers, which are color-coded based on the family of transporters: solute carrier (purple), ABC eukaryotic-like (pink), phosphotransferase (orange), major facilitator superfamily (yellow), ABC prokaryotic-like (green), and Others (blue). A heatmap display accompanies these data to show the strains and their values in direct comparison to the wild-type. One-way ANOVA was performed using Dunnett’s multiple-test correction. *P < 0.05, **P < 0.01, ***P < 0.001, ****P < 0.0001.

When investigating the fitness of genes in the murine model via Tn-seq, we are unable to discern which transposon mutants may have generalized growth deficiencies from those with true host colonization defects. Therefore, to better delineate these two aspects of pathogenesis, we individually cultured all 466 transposon mutants in both LB and human urine in vitro as a proxy to assess growth (Fig. 3). We evaluated three measures of potential growth deficiencies, area under the curve (AUC), final saturation optical density (OD_600_), and doubling time, and then performed statistical analysis to compare the parameters for each mutant against parent strain *E. coli* CFT073. We found that most mutants generally did not display significant growth defects in either medium. Although 21/466 (4.5%) mutants achieved statistical significance in growth media screens, these defects were relatively mild. 57% of mutants displaying growth deficiencies in LB were from the ABC prokaryotic-like family (Fig. 3*A*); however, none of these same mutants were significantly different than the wild-type in human urine. Two systems, Fep and Ycb, had defects in multiple genes of the operon. Due to both the variation in batch-to-batch human urine and the low saturating OD, achieving statistical difference becomes more difficult. Only 4 of the 21 growth-deficient mutants in LB also had significant defects in human urine, and only two of these (*zupT* and *panF*) were urine-specific growth deficiencies (Fig. 3*B*). Seven of the growth-deficient mutants overlapped with fitness defects in vivo (Fig. 2) and of these, five were in the ABC prokaryotic-like family. This means that 14 mutants that had a detectable in vitro growth defect (Fig. 3 and Dataset S3) did not display an in vivo defect.

### Surveying Growth on Murine Organ Agar Reveals Additional Growth and Nutrient Acquisition Deficiencies.

A recently developed model tested in our lab with uropathogen *P. mirabilis* indicates that growth on murine organ agar serves as an excellent proxy in vivo fitness (38). By homogenizing murine bladders, kidneys, livers, and spleens, it is possible to make solid agar plates. The only nutrients the bacteria can utilize are the ones from the homogenized organs. Mutants were spotted on the agar and incubated overnight. We then utilized image capture and subsequent densitometry of the images to determine growth defects. We plotted the number of pixels in each culture spot to identify outliers with defects in growth density (*SI Appendix*, Fig. S5 *A*–*D*).

**Fig. 4.**
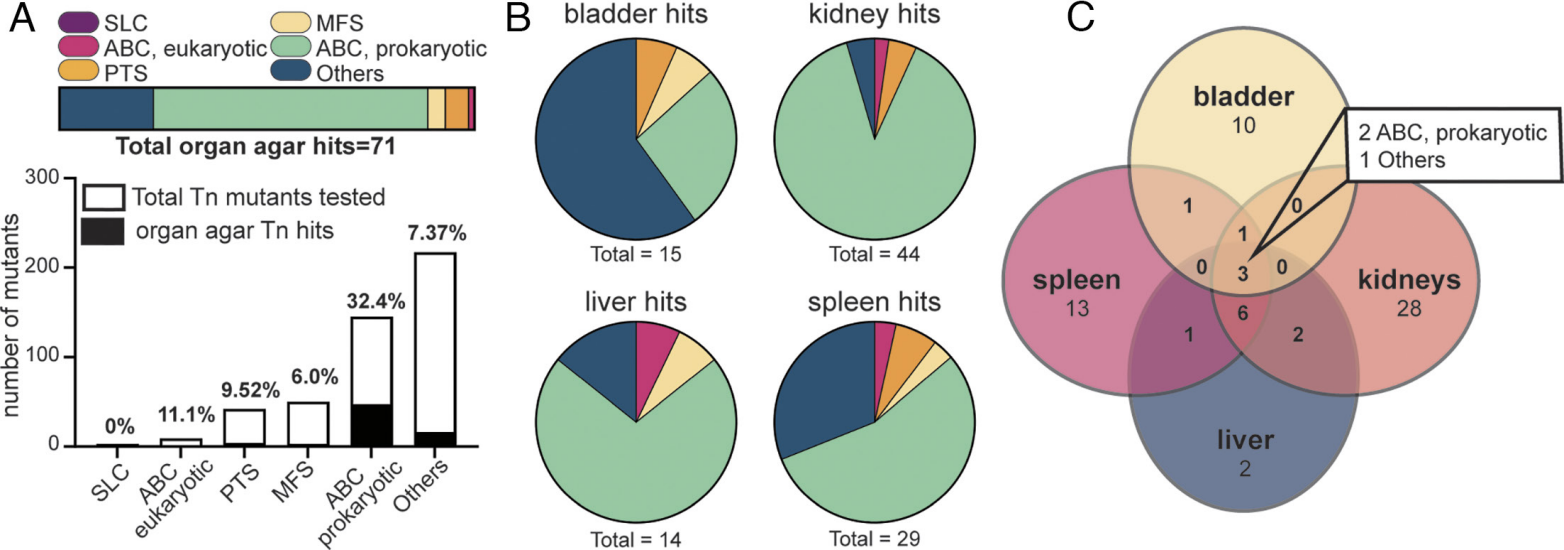
Screening of transposon mutants on murine organ agar ex vivo. All 466 Tn mutants were tested for growth on agar solely containing one of four homogenized mouse organs including bladder, kidneys, liver, or spleen. (*A*) A total of 71 mutants displayed growth defects on one or more types of organ agar. The families of those transporter mutants are indicated as a percentage of the total by color in the horizontal bar: solute carrier (purple), ABC eukaryotic-like (pink), phosphotransferase (orange), major facilitator superfamily (yellow), ABC prokaryotic-like (green), and Others (blue). The total number of transposon mutants tested from each family is indicated by the height of the entire bar. The percentage of mutants that were detected as hits is indicated above the bar in black. (*B*) Each type of organ agar had a different number of fitness factors, as indicated under the pie chart. The family of each transporters hit is also denoted in the pie chart by color. (*C*) The Venn Diagram visualizes overlap among the individual organ agars. Three mutants had defects on all four organ agar types, and their respective families are listed.

As with in vivo Tn-seq results, we found that most of the hits (66.2%) were in the ABC prokaryotic family (Fig. 4*A*). Interestingly, we observed a very different profile on bladder agar as compared to agar made with homogenized kidneys, livers, and spleens, with the Others family being more represented (Fig. 4*B*). Two out of three hits detected with inhibited growth on all four organ agar types were from the ABC prokaryotic family of transporters (Fig. 4*C*). This leaves 115 mutants still defined as infection-specific (*SI Appendix*, Fig. S6 and Dataset S4), having a defect in Tn-seq but not in either growth screen (24% of total mutants tested). By examining overlap from the organ agar defects (n = 71) from those determined to have in vivo disadvantages (n = 145), we can again assess a different category of mutants that we determine to have host-specific nutritional deficiencies: There are 23 mutants that fall into this category. Forty-four mutants had a defect on one or more organ agars, but not in vivo or during liquid growth (*SI Appendix*, Fig. S6 and Dataset S4). Overall, 43.6% of mutants tested had a defect in at least one screening experiment.

### The Family of ABC Transporters Contributes to In Vitro Urovirulence Phenotypes.

**Fig. 5.**
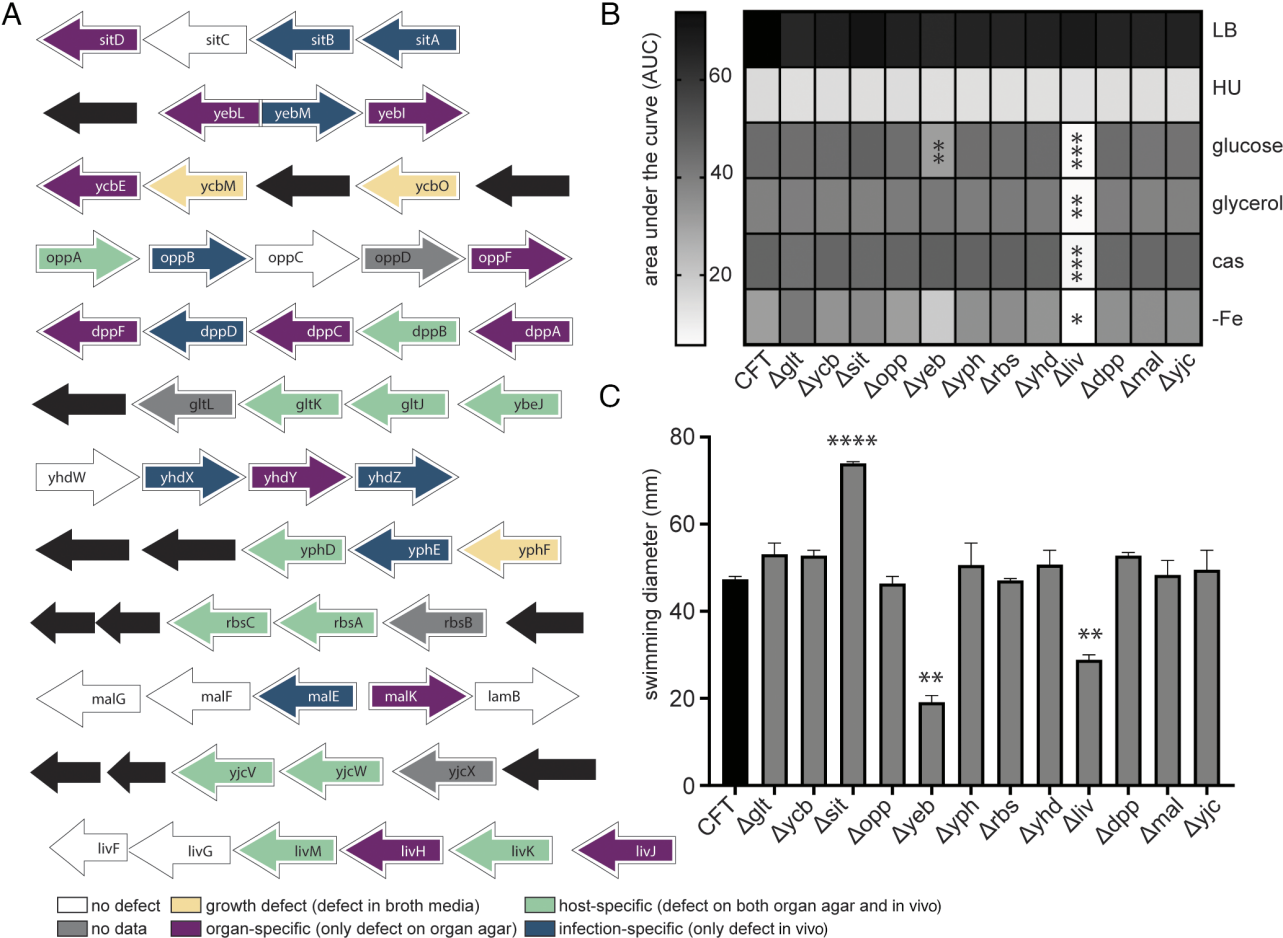
ABC transporter mutants dominate as fitness factors both in vivo and ex vivo and result in unique in vitro phenotypes. (*A*) Twelve ABC operons were found to have multiple mutants displaying either infection-specific (blue), host-specific (green), organ-specific (purple), or growth (yellow) defects. *SI Appendix*, Fig. S6 has a flow diagram for Tn mutant binning and Dataset S4 has all the binning assignments for each mutant. These entire transport systems were deleted using Lambda Red Recombinase. (*B*) These mutants were tested for growth in LB, human urine (HU), and MOPS supplemented with glucose (0.2%), glycerol (0.2%), or casamino acids (0.4%) as a sole carbon source. An iron-free MOPS medium containing glucose was also tested with 0.2% glucose. Area under the curve was determined for each mutant in all media formulations and is displayed on the heat map. One-way ANOVA was performed using Dunnett’s multiple-test correction compared to WT CFT073. *P < 0.05, **P < 0.01, ***P < 0.001. (*C*) Mutants were also tested for motility in semisoft (0.25%) agar. Following incubation at 30 °C, swim diameters (mm) were measured. One-way ANOVA was performed using Dunnett’s multiple-test correction compared to WT CFT073. **P < 0.01, ****P < 0.0001.

We now have defined transporter defects based on three separate screening methods. We observed that ABC prokaryotic transporters were the overrepresented family responsible for detected deficiencies (Dataset S4). Additionally, this family is most widely conserved across uropathogenic species (Fig. 1*C*). Thus, we prioritized these transport systems for further study based on those that had multiple defects within the same operon as determined by screening methods. There were 12 ABC transporters in which multiple transposon mutants displayed significant defects, providing the highest confidence for validation (Fig. 5*A*). We proceeded to construct deletion mutants of these twelve systems in the parent strain using lambda red recombination and phenotypically characterized these mutants. Surprisingly, the branched-chain amino acid import mutant (*liv*) was unable to grow in a minimal medium (Fig. 5*B*). Similarly, this mutant had lost the ability to swim (Fig. 5*C*). This was unforeseen because *E. coli* CFT073 also can biosynthesize all the branched-chain amino acids and is therefore not auxotrophic (19, 39). Likewise, we detected the zinc uptake mutant *yeb* to have a defect in growth and motility (Fig. 5 *B* and *C*), previously documented by others (40). Overall, we conclude that the deletion of ABC transporters can also result in distinctive urovirulence phenotypes, such as altered growth and motility. These pleiotropic effects on downstream virulence presentation highlight the role of transport systems as indirect virulence factors through modulation of phenotypic behavior.

### Redundancy and Regulatory Compensation Prevent Strong Fitness Defects from a Single System Deletion In Vivo.

**Fig. 6.**
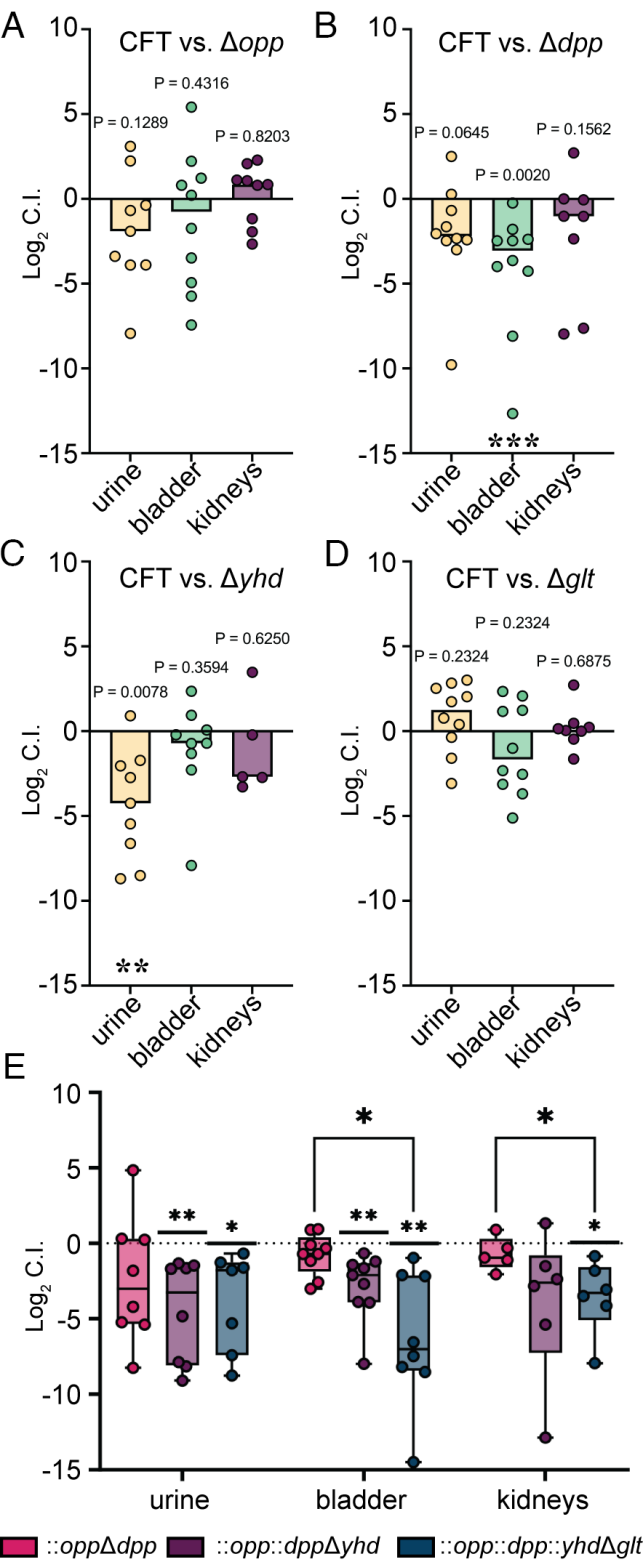
Substantial loss of fitness during infection requires multiple deletions in importers with overlapping functions. (*A*−*D*) CBA/J mice (n = 10) were transurethrally inoculated with a 1:1 mixture of *E. coli* CFT073 wild-type and the indicated mutant. Log_2_ Competitive Index (C.I.) was calculated for each organ site (mutant/wild-type). The dashed line at 0 signifies no deficiency for either the wild type (CFT) or mutant, a positive value indicates the mutant outperformed the WT, and a negative value defines the mutant as having a fitness defect. Wilcoxon-signed rank test was performed using a hypothetical mean of 0. *P* values are listed. (*E*) Three separate cochallenges were performed (n = 10, per mutant) in the same mouse model using a double (pink), triple (purple), and quadruple (blue) ABC importer mutants. Wilcoxon-signed rank test was performed using a hypothetical mean of 0. *P < 0.05, **P < 0.01. An additional mixed-effects analysis with Tukey’s multiple comparison test was used to determine differences among mutants within each organ site. *P < 0.05, **P < 0.01.

Next, our chosen ABC prokaryotic transporter deletion mutants were evaluated in vivo (*SI Appendix*, Fig. S7). During our Tn-seq experiments, significant defects (*P* < 0.05) ranged anywhere from −2.6 to −12.0 Log_2_ Fold Change (Dataset S3). To validate these findings, we used a cochallenge model with each mutant versus wild-type. Deletion of the entire peptide/amino acid transport systems led to a median fitness defect in bladders of mice (Fig. 6 *A*–*D*), but only Δ*dpp* was statistically significant in the bladder (Fig. 6*B*). The Yhd system, which is annotated as a general L-amino acid importer (17), had a significant defect in the urine and displayed the strongest defect of −2.69 Log_2_ FC in the kidneys (Fig. 6*C*). The glutamate/aspartate uptake system (Glt) only had a trending defect in the bladders (median −1.67 Log_2_ FC), and in the urine it outnumbered the wild-type (Fig. 6*D*).

Due to these relatively subtle defects seen with the deletion of only a single transporter, we proceeded to make double, triple, and quadruple mutants in compensatory peptide/amino acid importers. First, we generated a double knockout in the Opp and Dpp systems (41). Then, we added a third deletion in a nonspecific amino acid importer Yhd (42), followed by a glutamate-specific importer Glt. These three multigene knockout constructs were tested individually against the wild type in the murine model (Fig. 6*E*). We observed a steady decrease in the fitness of the mutants with each additive gene deletion going from −0.70 to −7.00 Log_2_ FC in the bladder (*P* = 0.0364) and −0.36 to −3.28 Log_2_ FC in the kidneys (*P* = 0.0315) (Fig. 6*E*). Therefore, additional mutations in redundant, and perhaps alternate, import systems decreased the fitness by 10-fold. Of note, only the quadruple mutant had a growth defect in minimal media with casamino acids as the sole carbon source (*SI Appendix*, Fig. S8). This suggests that an entire class of importers may need to be targeted in order to see therapeutic effects.

Since the original mutants tested were insertional transposon mutants into a single gene, the whole system deletion mutants are not a direct comparison to those that were originally screened. We, therefore, proceeded to make single subunit deletion mutants in select ABC importers *ycbO*, *sitA*, *yebL*, *yphF*, and *ybeJ* (*SI Appendix*, Fig. S9). These mutants were selected based on those displaying in vivo and in vitro defects (Fig. 5 and Dataset S3). Three of these mutants had better colonization in the mice than their whole system deletion counterparts (*SI Appendix*, Fig. S9 *A*–*C*). However, the *yphF* mutant performed similarly and the *ybeJ* was more defective than the comparable *glt* mutant (*SI Appendix*, Fig. S9 *D* and *E* and Fig. 6*D*). Likewise, we took both the *sit* and *yeb* mutants, which had motility defects in vitro and directly compared the single subunit versus the whole system knockout (*SI Appendix*, Fig. S9*F*). We found that these mutants performed differently than their respective whole system deletions. Therefore, we again emphasize the influence of these importers on subsequent virulence phenotypes but also suggest that having partially functioning transport systems can have detrimental cellular effects.

To probe this hypothesis further, we designed in vitro experiments to test the potential transcriptional redundancy of import systems. The *opp* and *dpp* mutants were grown in LB and human urine to measure the gene expression of related, functionally redundant defined import systems (41) (*SI Appendix*, Fig. S10). Relative to the wild-type strain, the two mutants displayed differential gene expression of alternate peptide/amino acid uptake systems. This up- or downregulation was dependent on the media type. For example, *dppA* was up-regulated in *opp* mutant in LB but mildly down-regulated in human urine (*SI Appendix*, Fig. S10*A*). A kinetic study in both growth media demonstrated varied temporal regulation of transporter genes in human urine relative to LB. Expression of ABC prokaryotic genes *oppA*, *dppA*, and *dppB* in wild-type peaked at 2 h in human urine, but 4 h in LB (*SI Appendix*, Fig. S10*B*). This contrasts with the MFS-like systems *dptC* and *dptD* which were consistently more up-regulated in human urine. Further examination of the temporal regulation of import systems showed a unique hierarchy with each medium, likely due to substrate availability and abundance. In LB, the ABC systems were consistently up-regulated up to 8 h (*SI Appendix*, Fig. S10*C*) but were down-regulated after the 2 h timepoint in human urine (*SI Appendix*, Fig. S10*D*). The DtpA and DtpB systems were unaltered in LB (*SI Appendix*, Fig. S10*C*) but up-regulated in human urine over time (*SI Appendix*, Fig. S10*D*). This is particularly interesting in light of transcriptional studies which demonstrate a higher correlation with bacterial growth in LB (in lieu of human urine) compared to patient infection (13).

## Discussion

UTIs are the second leading infectious disease worldwide. Over 90% of these infections are caused by Gram-negative bacterial species, with the dominant strain being uropathogenic *E. coli* (UPEC) (32). Strikingly, the growth rate of UPEC in women with active UTI is rapid with an average doubling time of 22.4 min (43). How the bacteria achieve such a fast growth rate in notoriously nutrient-poor conditions is not well understood, and yet, seems to be a consistent mechanism for achieving infection in vivo (10, 14, 43, 44). This study deployed unbiased in silico, in vivo, and in vitro screening approaches of bacterial transport systems to identify those most critical for host-specific growth and infection-specific colonization. The unique use of murine organ agar to determine the growth of individual transposon mutants allowed us to resolve host-specific nutritional deficiencies that affected bacterial growth. In parallel, we performed Tn-seq analysis that determined transport systems critical for successful host colonization. By analyzing these two datasets in tandem, we identified the ABC family of transporters as a critical group contributing to uropathogenesis in the ascending murine model of UTI. Collectively, we provide evidence that the ABC family of transporters is dominant in generating in vivo colonization and urovirulence phenotypes in UPEC.

Often residing in the gut as a commensal organism (45), UPEC strains colonize and infect the urinary tract using a suite of diverse virulence factors (12, 27). This variable genomic content results in extreme genetic heterogeneity among *E. coli* (10, 46). This, in combination with the abundance of different bacterial species that can cause infections, makes the generation of an effective universal treatment for UTIs challenging. Development of novel therapeutics is critical with antibiotic resistance on the rise (47). Creating new drugs for effective treatment against diverse populations of pathogens may include targeting of the core genome or overlapping vital cellular processes such as metabolism and transport. The benefit of targeting bacterial growth, instead of bactericidal treatments, is that it generates less selective pressure that may fuel the emergence of antibiotic resistance. Here, we propose that ABC prokaryotic-like transporters promote UPEC uropathogenesis and are a widely conserved potential target among UTI-causing bacterial species. Additionally, *E. coli* in the gut has been shown to rely on sugar substrates and glycolysis-mediated metabolic processes (48, 49), while *E. coli* in the urinary tract prefers peptide substrates to fuel gluconeogenesis and the TCA cycle (15, 50). Therefore, targeting of ABC transport, as opposed to sugar import PTS systems, may lead to decreased off-target effects to important commensal populations. Although ABC transporters are conserved across kingdoms, the ABC prokaryotic-like family possesses unique sequence and structure variation (20). Indeed, plants secrete compounds to inhibit bacterial ATP-binding transport systems (51) without harming their own, suggesting an increased likelihood for therapeutic success.

These transport systems are highly conserved among bacterial species due to their essential role in cellular processes, often residing in the core genome (14). However, for this same reason, bacteria encode several highly redundant functional homologs of these systems (12). Iron acquisition systems are a well-studied example of this phenomenon; for example, UPEC encodes five different iron acquisition structures and several have to be deleted to observe a loss in fitness (52, 53). Here, we demonstrate a 10-fold defect with a quadruple amino acid transport mutant (Fig. 6*E*), highlighting that multiple systems must be deleted for a substantial loss of fitness. This suggests a need to develop a broad inhibitor targeting the entire family of similar transporters. We also demonstrated a transcriptional compensation mechanism in which bacteria defective in oligopeptide transport (*opp*) up-regulate dipeptide import systems. The selective temporal preference for ABC transporters appears to be dependent on nutrient availability and growth phase, increased during early exponential (2 h) time points. The remarkably rapid growth rate of UPEC in patient urine (43) further supports this transporter family as a target for bacteriostatic treatments.

Slowing of bacterial growth via targeting import systems may have widespread consequences, as this will also inhibit essential metabolic processes. Iron is a host-limited and essential nutrient for bacteria, and amino acids have been determined as the preferred metabolic substrate for UPEC during UTI (15, 16, 30). Iron and amino acid import are both intimately tied to the TCA cycle, which is critical for UPEC fitness in vivo (15, 30). For example, enzymes in the aerobic TCA cycle that function under iron-limiting conditions are specifically important for uropathogenesis (54) and cytochrome *bd* is specifically critical for the anaerobic metabolism required in the intracellular niche (30). Indeed, iron uptake is up-regulated through ABC importer *sitABCD*, which has been demonstrated as important in this work and the work of others (55). Similarly, several amino acid-based metabolic processes are under selective pressure during infection (56). It is therefore not surprising that UPEC would rely heavily upon oligo-, dipeptide, and amino acid transporters during UTI (15, 16). An exception to this may be during glycosuria in which UPEC shifts toward glycolytic metabolism; however, even in this condition, UPEC was found reliant on the methionine ABC uptake system (*metQN*) (57). This supports the necessity of ABC importers among a wide variety of infectious niches including rapid growth in planktonic populations, intracellular survival, and diabetic urine.

Now, we also have host nutrient-specific and infection-specific defects detected with several amino acid-based transport systems. This independent corroboration implicates similar findings. For example, OppA (oligopeptide) and DppA (dipeptide) transporter subunits are fitness factors for UPEC during UTI (15). In *Salmonella enterica* serovar *Typhimurium*, Dpp has been implicated in chemotaxis (23, 58) and proton-dependent peptide importers cause flagellar gene upregulation (59). Our findings differ in that we found nonproton motive force-driven transporters to be largely contributing to phenotypes, such as the growth and motility defects observed with loss of the Liv (BCAA) system. This system was similarly documented to contribute to UPEC fitness during bacteremia (60). Additionally, partial deletion or disruption of a polycistronic transport system may be differentially detrimental to virulence outcomes compared to a deletion of the whole system. For example, we did not see significant defects with deletion of the Opp system as was previously reported with an *oppA* mutant (15), which we hypothesize is due to differential deletion techniques causing dissimilar energetic and compensation mechanisms. Indeed, we also documented several instances of single gene transport mutants behaving differently than entire transport system mutants. Future work will aim to understand the cellular energetics underpinning these phenomena. This observation makes ABC transport systems as a target for therapeutics even more appealing: Targeting a single component of the system may be a more effective inhibitory outcome.

Our observations have broader relevance and impact. For example, the use of organ agar screening methods can be used with a variety of host-pathogen combinations, even with in vivo models that have physiological or genetic limitations (38). Our work sheds light on the critical role of ABC transporters in UPEC’s survival in the host nutrient niche, host colonization, and subsequent altered phenotypes. Specific transporters from the ABC family have been documented as important in other pathogens as well, including *Streptococcus pneumoniae* (61), *S. Typhimurium* (62), and *Neisseria meningitidis* (63) among others. This study is unique in that we leveraged our transposon mutant library to undertake massive, unbiased screening approaches. These data provide a platform for future mechanistic studies into the systems that fuel uropathogens. This large dataset is now available as a resource for the uropathogen and the greater bacterial pathogen community for 1) further pursuit of genes of interest and 2) to serve as a model system for screens in other pathogens. Ultimately, this work contributes to the current knowledge gap that exists between bacterial nutrient acquisition and pathogenesis.

## Materials and Methods

### Bacteria Growth, Culture, and Mutagenesis.

*E. coli* CFT073 was regularly cultured in low-salt (0.5%) Luria broth with aeration at 37 °C overnight. Mutants containing transposon insertions were selected from an ordered library (10) and maintained in a medium containing kanamycin (25 µg/mL). All strains are reported in *SI Appendix*, Table S1. Isogenic mutants were constructed in strain CFT073 using the lambda red recombinase system (64). Primers used are listed in *SI Appendix*, Table S2. Once confirmed, mutants were maintained in a medium containing antibiotics (25 µg/mL kanamycin or 20 µg/mL chloramphenicol) during overnight culture but not during assays or growth screens.

### Ascending Murine Model of UTI.

Female CBA/J mice (aged 6 to 8 wk) were transurethrally inoculated with 2 × 10^8^ CFU suspended in PBS, as previously described (65, 66). See *SI Appendix*, *Supplemental Methods* for more details.

### Sequencing Preparation and Analysis.

Genomic DNA from bacterial lawns was isolated using the Wizard kit (Promega) and sheered with sonication. The resulting fragmented DNA was prepped for Illumina sequencing as previously described (10). See *SI Appendix*, *Supplemental Methods*.

### Bioscreen-C Growth Curve Analyzer.

All 466 transposon mutants and lambda red mutants were screened for growth defects in multiple media types. Overnight cultures were added to fresh media at a 1:100 dilution. Incubation was conducted over 20 h with aeration at 37 °C in the Bioscreen-C for OD_600_ readings every 15 min. MOPS minimal medium was made with either 0.2% glucose, 0.2% glycerol, or 0.4% casamino acids as sole carbon source. Human urine was collected from six or more healthy donors, pooled, and filter sterilized before use. See *SI Appendix*, Supplemental Methods for growth parameter calculations.

### Stamping on Organ Agar.

Organ agar was made from homogenized murine bladder, kidneys, liver, and spleen of female CBA/J mice (aged 6 to 8 wk) as described previously by us (38). See *SI Appendix*, *Supplemental Methods* for more detailed procedure.

### PATRIC Data Analysis.

Protein FASTA sequences of 640 KEGG annotated transporters in CFT073 were examined using BLAST against whole genomes of 1) *E. coli* isolates from human feces (n = 93), 2) enterohemorrhagic *E. coli* isolates (n = 139) from 3) UPEC (n = 457), and 4) type strains of other UTI pathogens available on BV-BRC. See *SI Appendix*, *Supplemental Methods* for BLASTP parameters and Dataset S1 for the list of *E. coli* genomes used.

### Bacterial Swimming Assay.

Overnight cultures of bacteria were normalized to an OD_600_ of 1.0 in 10 mM HEPES, pH 7.4. A sterile inoculating needle was used to introduce bacteria to the center of a semisolid agar plate (0.25%). Incubation took place at 30 °C for 16 h, then the diameter of the swim zone was measured in mm.

### Isolation of RNA and qRT-PCR.

Overnight cultures of bacteria were back diluted in choice media 1:100 and incubated shaking at 37 °C for the indicated time points. RNA collection, isolation, cDNA synthesis, qRT-PCR, and data analysis are described in *SI Appendix*, *Supplemental Methods*.

## Supplementary Material

Appendix 01 (PDF)

Dataset S01 (XLSX)

Dataset S02 (XLSX)

Dataset S03 (XLSX)

Dataset S04 (XLSX)

## Data Availability

Tn-sequencing data have been deposited in SRA (PRJNA964439) (67). All other data are included in the manuscript and/or supporting information.
