# Supplementary material for: Emerging roles for ABC transporters as virulence factors in uropathogenic Escherichia coli
Source: Proc Natl Acad Sci U S A. 2024 Apr 12;121(16):e2310693121. doi: 10.1073/pnas.2310693121 (PMC11032443; doi:10.1073/pnas.2310693121)
Supplement: Supplementary file 1 — Appendix 01 (PDF) [file pnas.2310693121.sapp.pdf]

Supplemental Materials and Methods

Bioinformatic data analyses. Briefly, using PATRIC/BV-BRC (1, 2) performed BLASTP using FASTA formatted amino acid sequences with a maximum of 5000 hits and E value threshold set to 0.0001. A protein sequence similarity of >90% was required to indicate the presence of an encoded protein against other *E. coli* strains. The exact genomes used for this analysis are listed in **Dataset S1** and were previously culled, used and validated (3). The odds ratio was determined by the following formula. The enrichment was a simple normalization of the odds ratio to the number of total genomes in each group being analyzed.

$$odds\ ratio = \frac{\text{UPEC}\left[\frac{\text{gene present}}{\text{gene absent}}\right]}{\text{fecal}\left[\frac{\text{gene present}}{\text{gene absent}}\right]}$$

Proteome comparison tool was set with a homology of >70%, minimum coverage of 30%, minimum identity of 10%, E value 1×10^{-5}. Prototype strains were used for each bacterial species: *Klebsiella pneumoniae* KPPR1 (4), *Proteus mirabilis* HI4320 (5), *Serratia marcescens* UMH9 (6), *Citrobacter freundii* UMH14 (7), *Providencia stuartii* BE2467 (8), *Acinetobacter baumannii* AB0057 (9), and *Escherichia coli* UTI89 (10). These strains were selected because they had existing transposon studies and well-annotated genomes.
KEGG annotations were based on KO 02000 classification and reference genome (11, 12). ABC classifications based on Igarashi *et al*. (2004) and Tomii & Kanehisa (1998) and MFS classifications were from TCDB (13).

Ascending murine model of UTI. For Tn-seq experiments, mutants were cultured statically overnight and OD_{600}-matched before pooling for inoculation into the mice. Each mutant pool was inoculated into 10-15 mice (**Fig. S4**). For co-challenge experiments, the inoculum was prepared with wild-type CFT073 and mutant mixed in a 1:1 ratio. Twenty-four hours post inoculation, urine was collected followed by an aseptic harvest of the bladders and kidneys. Bacterial colonization was determined by plating a sample of homogenized organs on LB agar plates and incubating overnight at 37°C. Differential plating on antibiotics was used for determining mutant *versus* wild-type CFU for co–challenge experiments. For Tn-seq experiments, the entire organ homogenate volume was spread-plated and all bacterial growth was collected the next day for genomic DNA extraction. The following formula was used to calculate $\log_2$ competitive indices (CI) for each mouse:

$$x = \frac{\left[\frac{CFU_{mutant}}{CFU_{WT}}\right] output}{[CFU_{mutant}/CFU_{WT}] input}$$

Sequencing preparation and analysis. Briefly, we performed blunt-end repair & dA-tailing of the fragmented DNA (NEB catalog #E7442S/L), followed by adaptor ligation with kit (NEB catalog #E7445S/L) and finally, transposon gDNA-junction enrichment using Phusion polymerase. DNA binding magnetic beads AMPure XP (Beckman Coulter, Agencourt catalog #A63880) were used for all DNA clean up steps. The final products were ethanol precipitated and assessed for quality by TapeStation. Normalization and sequencing were performed by

the University of Michigan Advanced Genomics Core. Fragments were sequenced on Illumina HiSeq4000 with paired end 150bp reads using 15% PhiX to increase sequence diversity. Sequencing reads were deconvoluted, trimmed and mapped using Bowtie to their location within the CFT073 genome (RefSeq NC_004431.1). Ratios of input to output reads were calculated and statistics for determining fitness contribution were performed as outlined in Goodman *et al*. (14, 15). Sequence reads are publicly available on the NCBI SRA (BioProject PRJNA964439).

Growth curve data analysis. Each growth curve during Tn mutant screening was analyzed for three different metrics: area under the curve (AUC), doubling time, and maximum OD_{600} for each biological replicate (n=3-5). Area under the curve was calculated by setting GraphPad Prism parameters to calculate with a baseline at the time zero OD_{600} for data up to 16 h. This same procedure was performed for lambda red mutant growth curve analysis. Doubling times (h) were calculated during exponential growth phase as defined by OD_{600} using the formula $r = \frac{(\ln\left(\frac{\mathrm{ODt2}}{\mathrm{ODt1}}\right))}{(\mathrm{t2}-\mathrm{t1})}$. These analyses were completed with GraphPad Prism 9.0 and a one-way analysis of variance (ANOVA) was performed using Dunnett's multiple-test correction to WT CFT073. Significance was defined as $P_{adj}<0.05$.

Stamping on organ agar. Briefly, organs were homogenized in 3mL sterile water and further diluted with sterile water 1:5 bladder, 1:10 kidney, 1:10 liver, 1:5 spleen, then mixed 1:1 with 2X molten agar cooled to 55°C. 20 mL agar was added to each 10 cm petri dish. Once solidified, the plate surface was air dried to prevent cross contamination in a biosafety cabinet. Bacteria cultures were stamped from overnight glycerol stocks on dry ice in a 48-well array using an ethanol-sterilized microplate replicator. Plates were incubated for 24 hours at 37°C and imaged using a Q count (Sprial Biotech) maintaining shutter speed and light source direction for each plate type. Images were processed in Adobe photoshop to remove color and apply an identical contrast filter to all images. Pixel intensity was calculated using Image Lab (Biorad) volume tools. A standardized round region was used for each bacterial colony in the array and local background subtraction applied. Strains with pixel intensity less than two standard deviations from the group mean for each organ type were identified as deficient for growth.

Isolation of RNA and qRT-PCR. Overnight bacterial cultures incubated in LB broth were back-diluted 1:100 into the appropriate medium type and cultured for the indicated time point. A 2:1 (vol/vol) ratio of RNA Protect (Qiagen) was added to 300 to 500 µL of bacterial culture and incubated at room temperature for 5 min. Samples were then pelleted and frozen at −80°C.

RNA isolation was performed with the RNeasy minikit (Qiagen) after treatment with lysozyme and proteinase K, and RNA samples were then DNase treated using a Turbo DNA-free kit (Invitrogen). The absence of DNA contamination was confirmed via PCR with CFT073 genomic DNA (gDNA) primers. 1 µg of RNA was converted to cDNA using an iScript kit (Bio-Rad). The resulting cDNA was diluted 1:50 in water and utilized for real-time quantitative reverse transcription PCR (qRT-PCR) in a QuantStudio 3 PCR system (Applied Biosystem) with SYBRGreen PowerUp reagent (Invitrogen). Samples were normalized to *gapA* transcript levels by subtracting

the comparative threshold cycle (CT) values of gapA from the values of monitored genes normalized to conditions indicated by the ΔΔCT method. Data were presented as the $\log_2$ fold change (FC) using the formulation $\log_2(2-\Delta\Delta CT)$.

Supplemental Figures

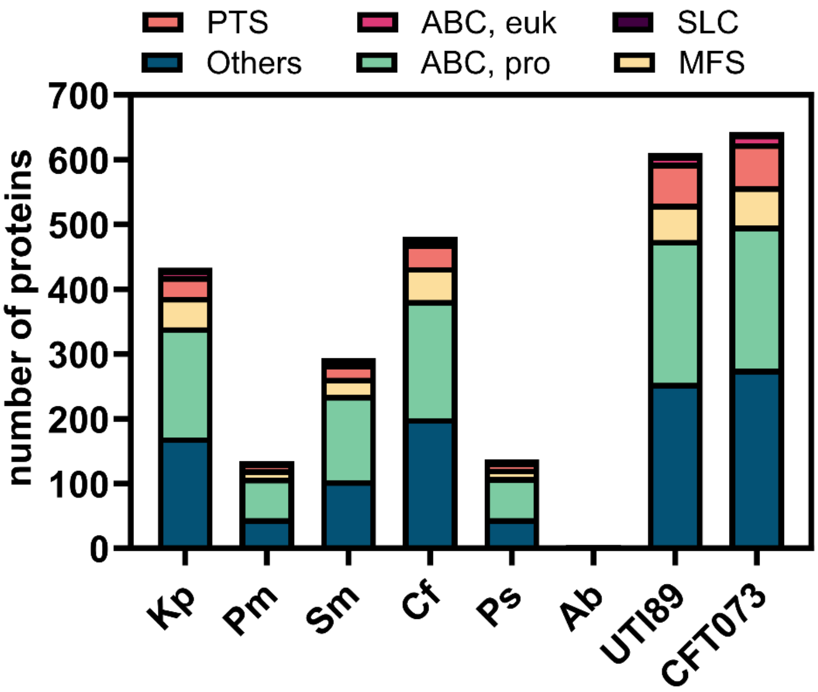

Figure S1. Conservation of UPEC transporters among across diverse uropathogens. The composition of total transport encoded proteins detected based on homology to *E. coli* CFT073 is displayed for each pathogen. BlastP parameters are described in more detail in the supplemental methods. Each color represents a specific transporter family. The graph is stacked, so total bar height is equal to the total number of CFT073 transporters detected in the respective pathogen genome.

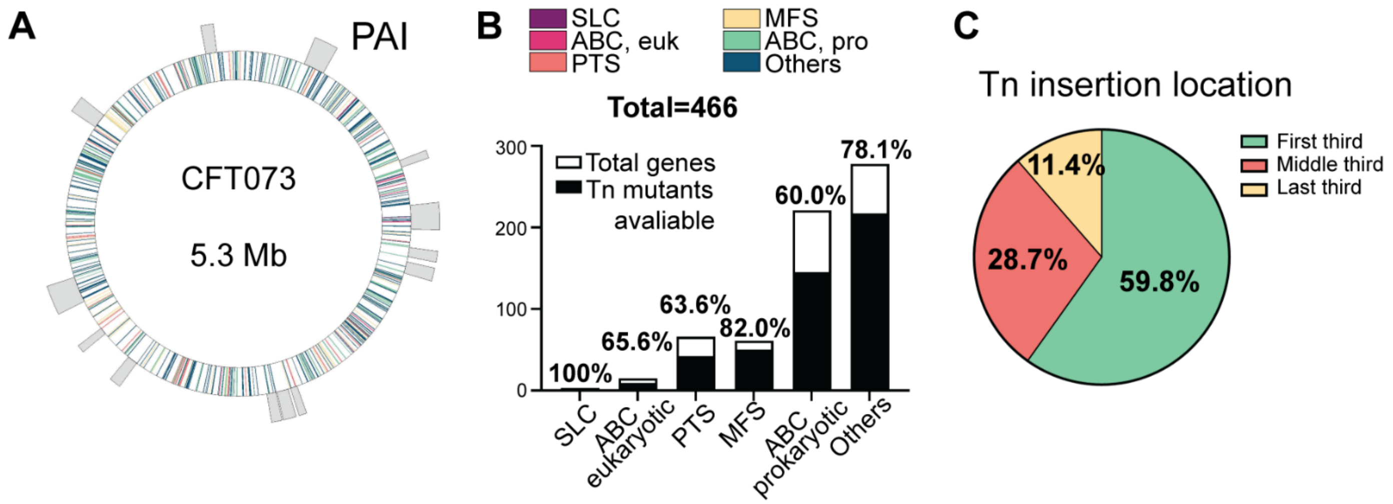

Figure S2. Transporters largely reside in the conserved genome and are available in the *E. coli* CFT073 ordered transposon library. (A) The genome of *E. coli* CFT073 is decorated with genes annotated as transport-related. Each of the six KEGG transporter families is marked with a unique color in the correct position on the genome: solute carrier (purple), ATP-binding cassette eukaryotic-like (pink), phosphotransferase (salmon), major facilitator family (yellow), ABC prokaryotic-like (green), others (blue). The grey areas indicate pathogenicity islands (non-core genome). (B) There are 640 KEGG predicted transport genes in the CFT073 genome, 466 (72.5%) of these were available as transposon mutants in the ordered library of CFT073 (10). The bar graph indicates the number of genes within each family (entire bar height) and the percentage of those that were available as mutants (black), exact percentages are designated above the bars. The linear color chart displays the number of transposon mutants representing each KEGG family. (C) The pie chart displays the proportion of the 466 mutants with the transposon insertion in the first (green), second (salmon), or last (yellow) third of the gene sequence.

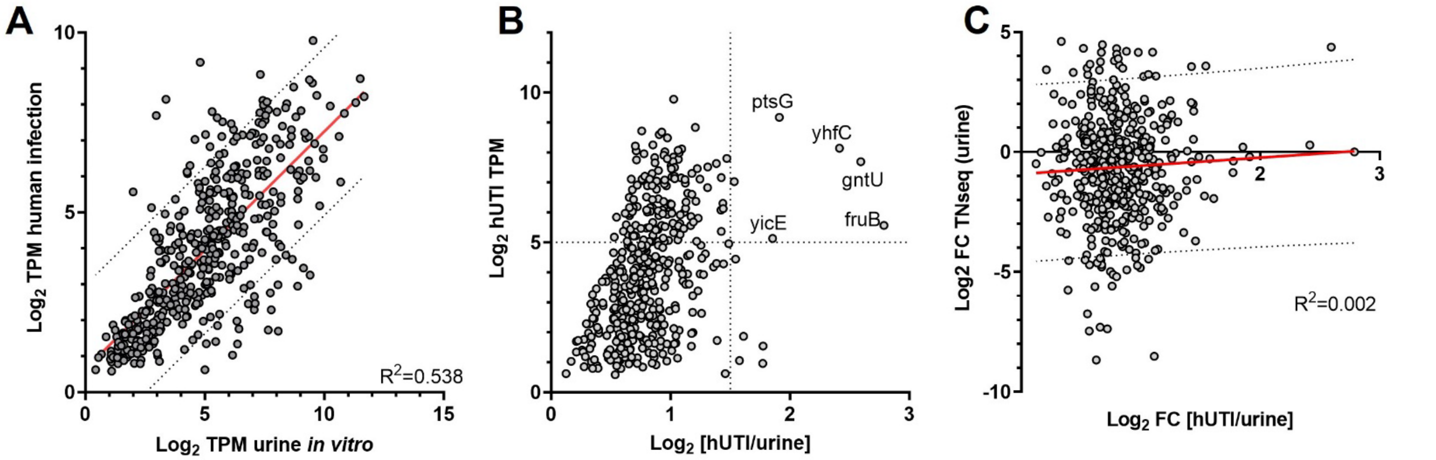

Figure S3. Gene expression of transporters in UPEC strains during UTI in women does not correlate with detected fitness *in vivo*. (A) Select transport genes expressed by UPEC in human patient urine *in vivo* during UTI *versus ex vivo* in urine from our existing dataset (n=453) (16). The correlation between the two was determined using a simple linear regression which produced a goodness of fit R^2 value of 0.538 that was significant P<0.0001. Each gray dot represents a single gene, the red line indicates the best fit, and the dashed lines denote the standard deviation. (B) The Log_2 fold change (FC) of reads mapped in urine during a UTI in women samples over urine *ex vivo*, is plotted of the x-axis. The vertical dashed line indicates 1.5 Log_2 fold-change increased expression in humans. The raw TPM (transcripts per million) values for each gene are plotted on the y-axis. The horizontal dashed line indicates a minimum of 5 reads. The upper right quadrant displays the genes that are the most highly expressed, and also up-regulated, in human patient urine. (C) Human urine *in vivo* gene expression during a UTI in women in TPM are plotted against obtained Tn-seq values, which produced a R^2 value of 0.002. Each gray dot represents a single gene, the red line indicates the best fit, and the dashed lines denote the standard deviation. All gene expression data are included in **Dataset 2**.

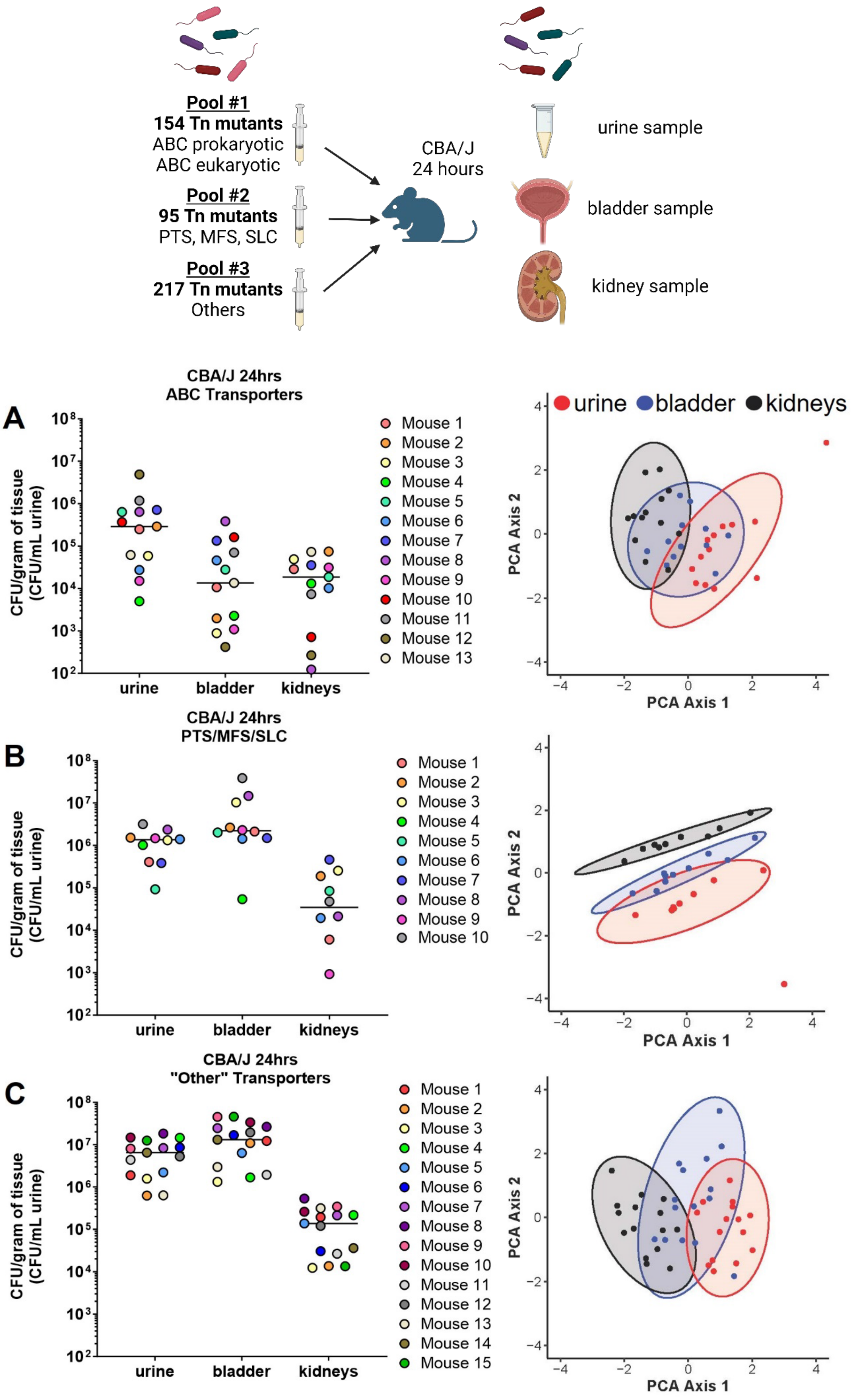
Pool #1
154 Tn mutants
ABC prokaryotic
ABC eukaryotic
Pool #2
95 Tn mutants
PTS, MFS, SLC
Pool #3
217 Tn mutants
Others
CBA/J
24 hours
urine sample
bladder sample
kidney sample
A
CBA/J 24hrs
ABC Transporters
CFU/gram of tissue (CFU/mL urine)
urine
bladder
kidneys
Mouse 1
Mouse 2
Mouse 3
Mouse 4
Mouse 5
Mouse 6
Mouse 7
Mouse 8
Mouse 9
Mouse 10
Mouse 11
Mouse 12
Mouse 13
urine bladder kidneys
PCA Axis 1
PCA Axis 2
B
CBA/J 24hrs
PTS/MFS/SLC
C
CBA/J 24hrs
"Other" Transporters
Mouse 14
Mouse 15

Figure S4. Colonization data and mutant fitness profiles in the murine model of ascending UTI. The CFU/g of tissue (or mL of urine) was determined. Each mouse is indicated by a unique color for easy tracking. The PCA plot takes into consideration the order of fitness factors (PC2), in addition to the magnitude in which they were defective (PC1) to visualize variance. These analyses were done for all three separate infection pools (A) ABC eukaryotic and prokaryotic mutants (n=154), (B) PTS, MFS, and SLC mutants (n=95), and Others (n=217).

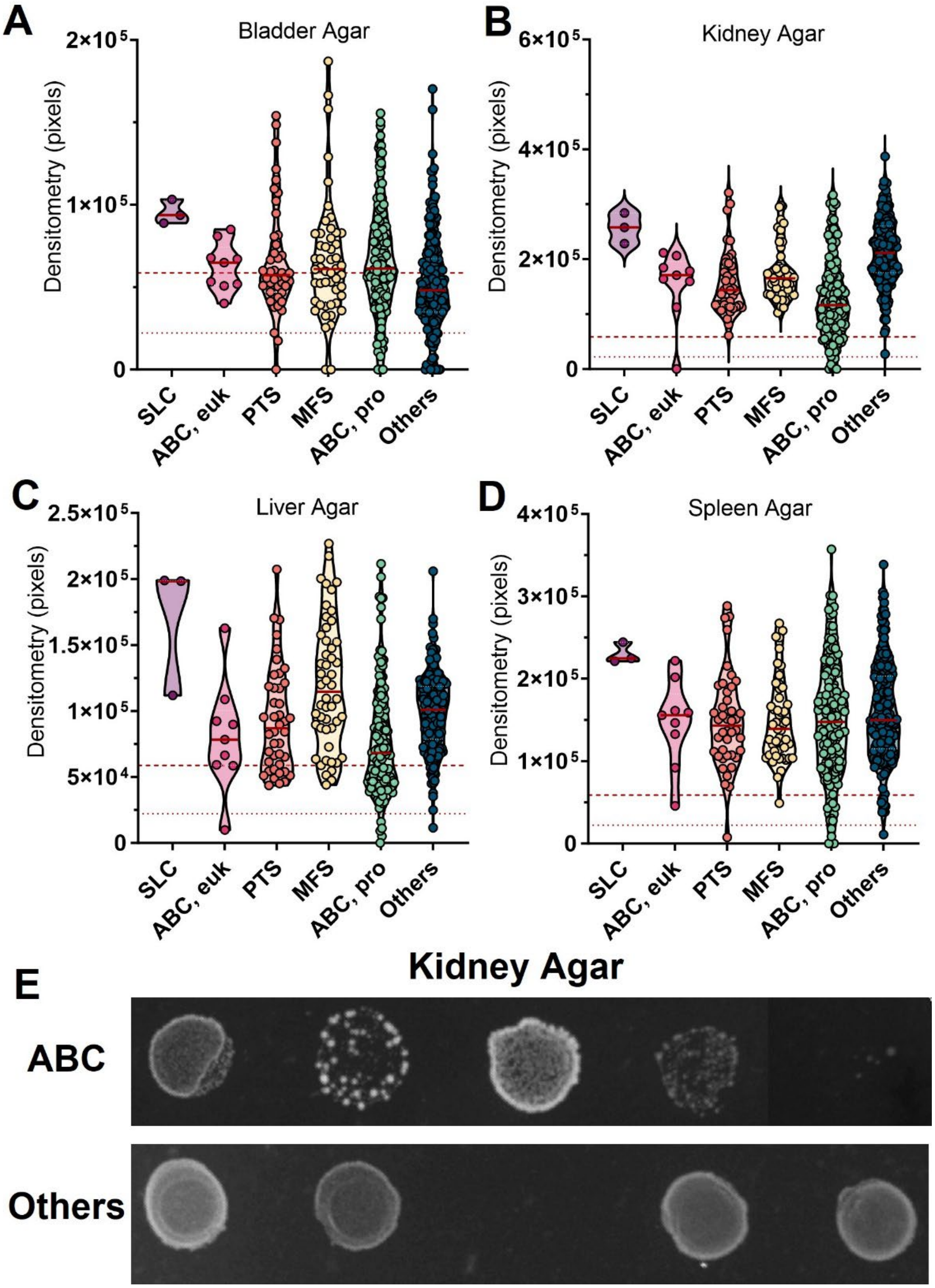

Figure S5. Densitometry on organ agar suggests different nutritional needs. All 466 transposon mutants were screened for growth on four types of agar made from the organ homogenates of naïve mice. Each dot represents a single mutant, the median for each family is the solid red line on (A) bladder, (B) kidney, (C) liver, and (D) spleen agar. (E) A representative image of spotting mutants on these plates is shown along the bottom of the figure for growth of mutants on kidney agar. Images were captured and densitometry was used to assess growth on the agar after 24 hours.

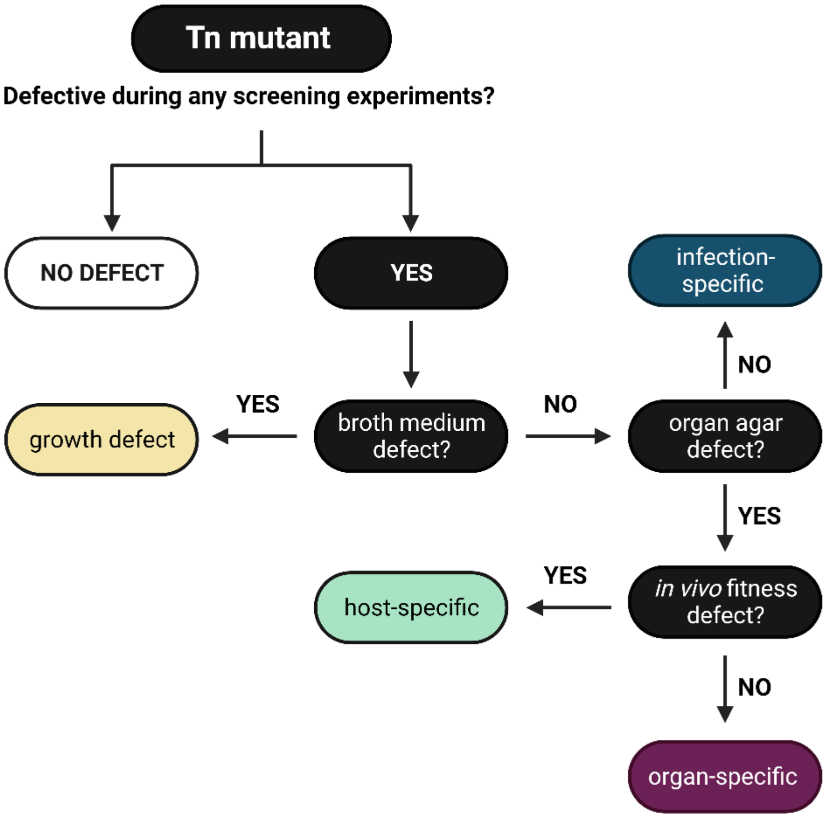

Figure S6. Decision tree for binning Tn mutants based on screening defects. All 466 transposon mutants were screened for growth *in vitro* (LB and human urine), growth on organ agar, and *in vivo* fitness in the ascending model of UTI. Any transposon mutant with a significant growth defect during *in vitro* broth testing was first binned (yellow). Those that did not have a growth defect in broth or *ex vivo* on organ agar, therefore only displaying a fitness defect *in vivo*, were deemed infection-specific (blue). Those that displayed a defect on organ agar, but not in vivo were classified as organ-specific (purple). Finally, those that had both a defect on *ex vivo* organ agar and *in vivo* were binned as being host-specific fitness factors (green).

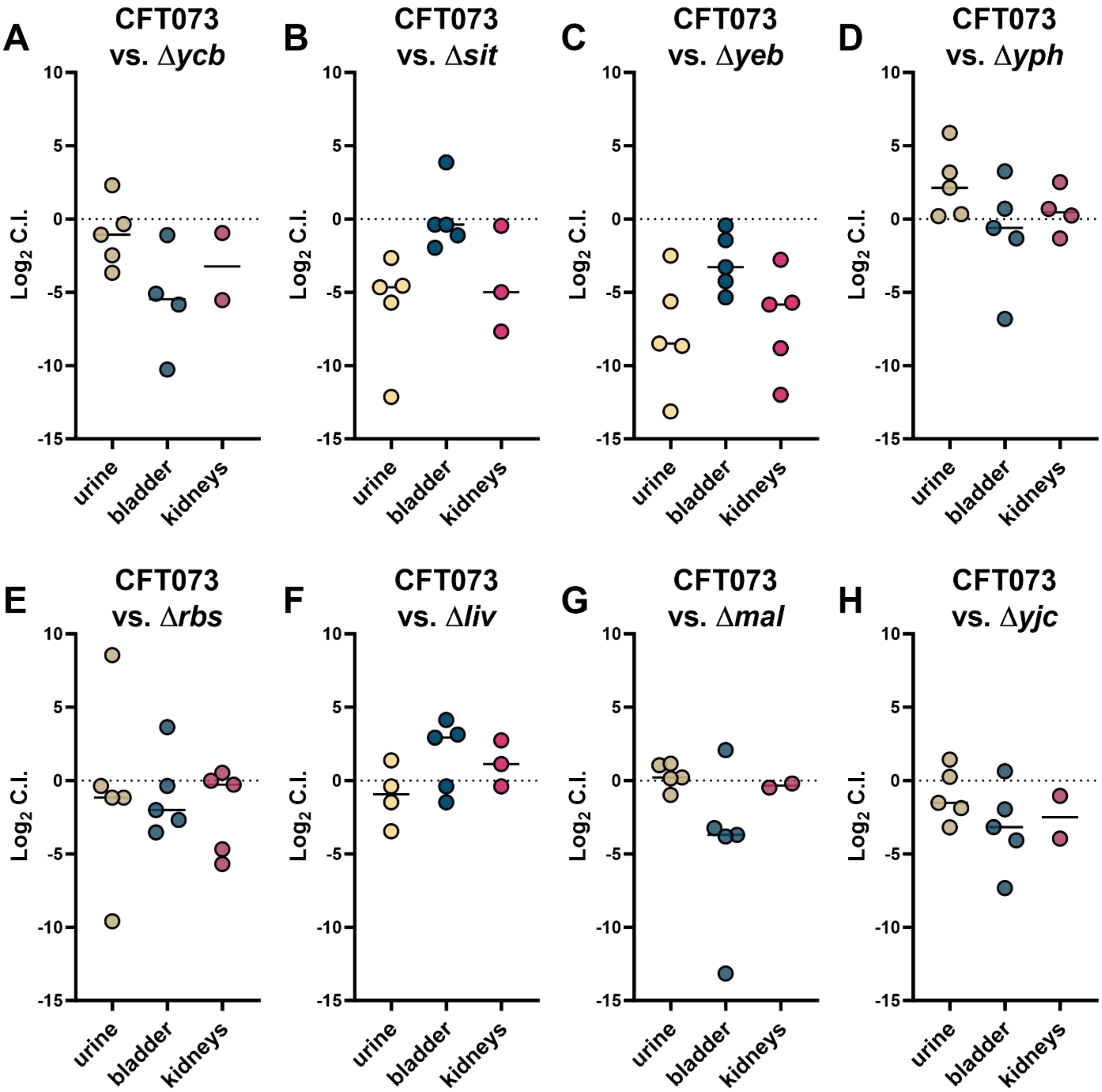

Figure S7. Murine co-challenge of select ABC importer mutants against wild-type. (A-G) CBA/J mice (n=5) were transurethrally inoculated infected with a 1:1 mixture of CFT073 wild-type and the mutant indicated. Log_2 Competitive Index (C.I.) was calculated (mutant/wild-type) for each organ site. The dashed line at 0 signifies no deficiency for either the wild-type or mutant.

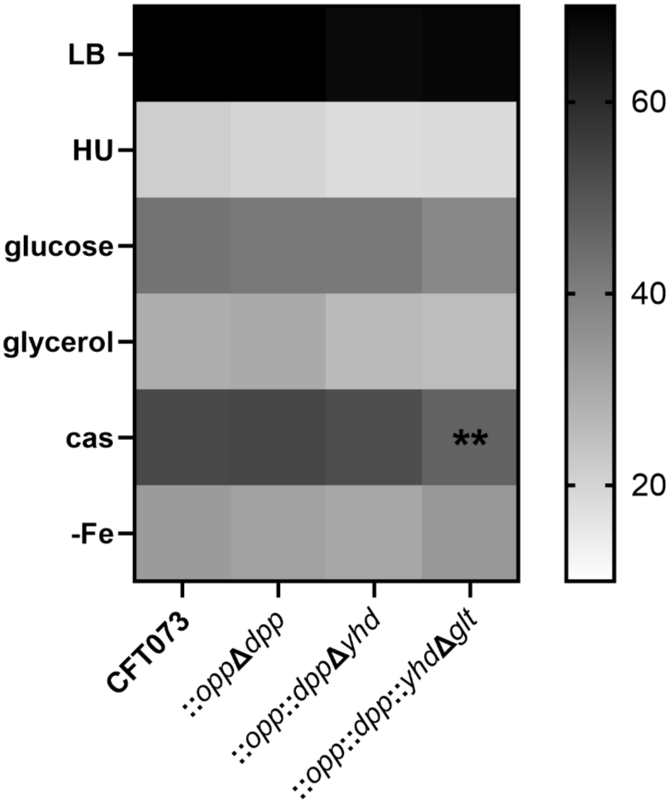

Figure S8. Phenotypes for double, triple, and quadruple transport mutants. These mutants were tested for growth in LB, human urine, and MOPS supplemented with glucose (0.2%), glycerol (0.2%), or casamino acids (0.4%) as a sole carbon source. An iron-free MOPS medium containing glucose was also tested with 0.2% glucose. Area under the curve was determined for each mutant in all media formulations and is displayed on the heat map. One-way analysis of variance (ANOVA) was performed using Dunnett's multiple-test correction compared to WT CFT073. **P<0.01.

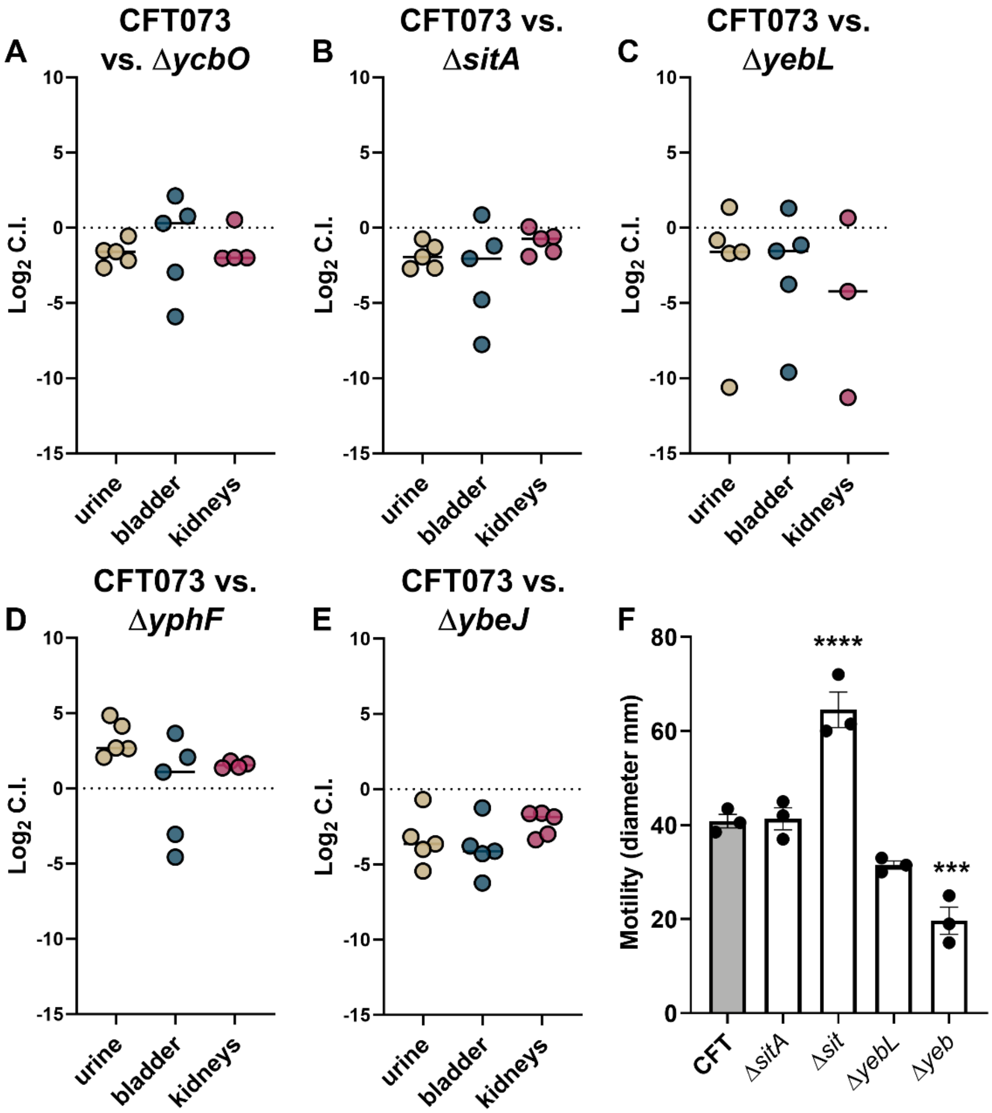

Figure S9. Single unit ABC knockouts produce different *in vivo* and *in vitro* phenotypes. (A-E) CBA/J mice (n=5) were transurethrally inoculated with a 1:1 mixture of *E. coli* CFT073 wild-type and the indicated mutant. Log_2 Competitive Index (C.I.) was calculated (mutant/wild-type) for each organ site. The dashed line at 0 signifies no deficiency for either the wild-type or mutant. (F) Select mutants were tested in a motility assay to compare the entire system deletion *versus* the single gene mutant. Dots represent the biological replicates (n=3), the bar height is the mean; error bars represent SEM. A one-way ANOVA was used to assess statical differences from wild-type CFT073. ***P<0.001; ****P<0.0001.

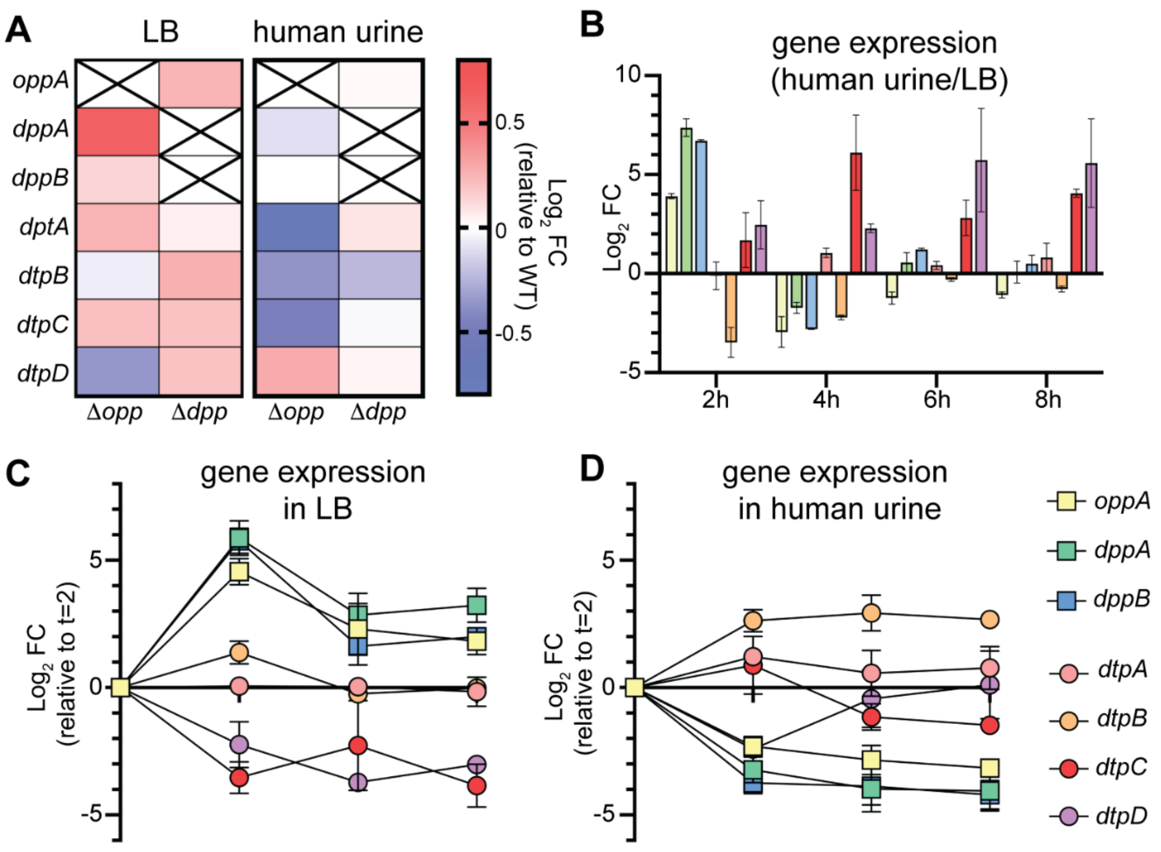

Figure S10. Transcriptional compensation in functionally redundant importers. (**A**) The wild-type, *opp*, and *dpp* mutant were grown at 37°C with aeration in LB or human urine and RNA was collected. qRT-PCR was used to determine the relative abundance of transcript in the mutant strains compared to the wild-type. Data are the mean of Log_2 fold change (FC). (**B**) RNA was isolated from wild-type bacteria at 2, 4, 6 and 8 h post-inoculation (n=2). The transcript regulation in human urine was normalized to that in LB at each time point to determine kinetic transporter gene expression. The bars indicate the mean and error bars are SEM. Time course expression in (C) LB and (D) human urine was measured over time and is shown relative to time 2 h. The symbols indicate mean and the error bars are SEM (n=2).

Supplemental Tables

Table S1: Bacterial strains used in this study		
Strain	**Genotype/Description**	**Reference/Source**
CFT073 (WT)	*E. coli* strain isolated from a patient with acute pyelonephritis	PMID: 2182540
Δ*glt*	CFT073Δ*glt*, Kan^r	This study
Δ*ycb*	CFT073Δ*ycb*, Kan^r	This study
Δ*sit*	CFT073Δ*sit*, Kan^r	This study
Δ*opp*	CFT073Δ*opp*, Kan^r	This study
Δ*yeb*	CFT073Δ*yeb*, Kan^r	This study
Δ*yph*	CFT073Δ*yph*, Kan^r	This study
Δ*rbs*	CFT073Δ*rbs*, Kan^r	This study
Δ*yhd*	CFT073Δ*yhd*, Kan^r	This study
Δ*liv*	CFT073Δ*liv*, Kan^r	This study
Δ*dpp*	CFT073Δ*dpp*, Kan^r	This study
Δ*mal*	CFT073Δ*mal*, Kan^r	This study
Δ*yjc*	CFT073Δ*yjc*, Kan^r	This study
Δ*opp*Δ*dpp*	CFT073::*opp*Δ*dpp*, Kan^r	This study
Δ*opp*Δ*dpp*Δ*yhd*	CFT073::*opp*::*dpp*Δ*yhd*, Cam^r	This study
Δ*opp*Δ*dpp*Δ*yhd*Δ*glt*	CFT073::*opp*::*dpp*::*yhd*Δ*glt,* Cam^r	This study
Δ*ycbO*	CFT073Δ*ycbO*, Cam^r	This study
Δ*sitA*	CFT073Δ*sitA*, Cam^r	This study
Δ*yebL*	CFT073Δ*yebL*, Cam^r	This study
Δ*yphF*	CFT073Δ*yphF*, Cam^r	This study
Δ*ybeJ*	CFT073Δ*ybeJ*, Cam^r	This study

Table S2: Primers used in study

Gene or Plasmid	Forward Primer	Reverse Primer
glt[a]	CCTAACAAACACAACACTGCACAATAAAGTCGCAGGTGTAGGCTGGAGCTGCTTC	AGATGGCGCGCAATTGAGTATGCGCGCCAGAGTGAATGGGAATTAGCCATGGTCC
ycb[a]	GCTAGAAACTTTCTGGCAGGCGTTGCACCGCCGCGGTGTAGGCTGGAGCTGCTTC	TGAAAATGCTGGTTTTGTGCGCCACATCCGGCATAATGGGAATTAGCCATGGTCC
sit[a]	TTTGTTCTAATTTTGCTCACTATAGGTACTAAATTGTGTAGGCTGGAGCTGCTTC	AAAAGTATCGGGTGTTGCTGTCCTCGGATGTTGTGCATGGGAATTAGCCATGGTCC
opp[a]	ATAAAGTAACCTGACAGCAGAAAGTCTCCGAGCCTGTGTAGGCTGGAGCTGCTTC	GAATAAGGGCTGACAACTGTCAGCCCTTATTGCTTATGGGAATTAGCCATGGTCC
yeb[a]	GAGCGGGCTATCTGTTGCACGTAATCACTTCCTCGTGTAGGCTGGAGCTGCTTC	TCGCGCCGCATCCGACAAATGTGTTCAGCGACAGAATGGGAATTAGCCATGGTCC
yph[a]	AAGCCGTTGTAAATAAAAACCCTACAGGAGGCACTGTGTAGGCTGGAGCTGCTTC	CATCCCTGCCGCACCCGCGCCAGGGGCGTTAAGGTATGGGAATTAGCCATGGTCC
rbs[a]	ATTTAAGACGTTGTACCCTACAAAAATAATGAGGAGTGTAGGCTGGAGCTGCTTC	AAGACCTTACTCATATGATTAATTCCTTGAAATGAATGGGAATTAGCCATGGTCC
yhd[a]	CCTATAACGATAATTTTCGCCTCACAGGAAGCATTGTGTAGGCTGGAGCTGCTTC	AGGATGGCCTTTTTGCTTTTCGAACTAACATTCAAATGGGAATTAGCCATGGTCC
liv[a]	TAAAGCACGGGTAGCTACGTATAAAACGAAATAAAGTGTAGGCTGGAGCTGCTTC	AAGGAGTCTTTTGACTCCTTATCAATCAACGTGTTAATGGGAATTAGCCATGGTCC
dpp[a]	GTTAAAACAACAAACATCACAATTGGAGCAGAATAGTGTAGGCTGGAGCTGCTTC	GAGTAAACCGGAGCGCATGGCCCCGGTTTTGTGAGATGGGAATTAGCCATGGTCC
mal[a]	GGGTGCAGCGGCATAACATTGGCAGAACAACATCTTTGTGTAGGCTGGAGCTGCTTC	CACCTGTGACAGGCTTTGTGTGTTTTGTGGGGTGCATGGGAATTAGCCATGGTCC
yjc[a]	CATCGCGGAGTTACCTCTCCCCTTACCGTGAGAACGTGTAGGCTGGAGCTGCTTC	ACATTAACGAGGGGGAGATTTTCATGTTCAATTCCATGGGAATTAGCCATGGTCC
ycbO[a]	GCTAGAAACTTTCTGGCAGGCGTTGCACCGCCGCGGTGTAGGCTGGAGCTGCTTC	ACCGTGGGTCGGTAAAAACCAGAACATATTCAGACATGGGAATTAGCCATGGTCC
sitA[a]	TTTGTTCTAATTTTGCTCACTATAGGTACTAAATTGTGTAGGCTGGAGCTGCTTC	CCAGCTTTAATTCCCTGTCCAGCGTCTGGTAGTACCATGGGAATTAGCCATGGTCC
yebL[a]	GAGCGGGCTATCTGTTGCACGTAATCACTTCCTCGTGTAGGCTGGAGCTGCTTC	GAAACCGAGACATTTTCCAGGGAAACCAGACTTGTATGGGAATTAGCCATGGTCC
yphF[a]	AAGCCGTTGTAAATAAAAACCCTACAGGAGGCACTGTGTAGGCTGGAGCTGCTTC	TTGCCGTGAACATAGGGACCTCTGCGAATCAGCGAATGGGAATTAGCCATGGTCC
ybeJ[a]	CCTAACAAACACAACACTGCACAATAAAGTCGCAGGTGTAGGCTGGAGCTGCTTC	GAGAGGGCTGGAATTTCCGCCCCTGGTTCTTGTAAAATGGGAATTAGCCATGGTCC
glt validate[b]	TGTAAGCAATGAAATCAGCCGC	CGAGGGCGAGAACTATTGC
ycb validate[b]	GGTCGATTATGCCCTTAAGCC	TCTTATCAGGCCTACAAGTCCG
sit validate[b]	GAGAGGTGCAAATCCTCCCATAC	GCTGGATTGAAATGTTCGGGC
opp validate[b]	CGCCAGGCGATTCTTTATTGG	GCCGAAGTGGTGATTGGTC
yeb validate[b]	TCGCGGTGATAAACATAGGGC	GCCAGAAATGCCGTAAGTCG
yph validate[b]	TGCTCGCGCTGAATTGATGG	GATCAGTACCTGGTTAATCCCCGG
rbs validate[b]	CAATCCTGCGGCAAATGATCC	TAATGATGCCGCACGGAACG
yhd validate[b]	AACTCAGAAGTGAAACGCCG	TCCATTTTCGCACCATTGCG

liv validate[b]	AATAATCAATTCCCCCTCCGGC	TTATCCGGCCTACAAGATCGTGC
dpp validate[b]	CTGTTGACAGATTGTAGGTCACG	GTTCTTTCAGTTGGGAAGCCC
mal validate[b]	GTCGATCATAAATCGCCACTGG	ATAGCCGTGGAAATCAACAGC
yjc validate[b]	ACAACTGACTTTGCTGGACGC	TCAGATTCGGCACAAAGTGGC
ycbO validate[b]	GGTCGATTATGCCCTTAAGCC	GCAACGGTAGGTGAGGTTACG
sitA validate[b]	GAGAGGTGCAAATCCTCCCATAC	TGACTGAGGGACGTAGGCAACC
yebL validate[b]	TCGCGGTGATAAACATAGGGC	TGTCGAGATACAGCTTCTGCGG
yphF validate[b]	TGCTCGCGCTGAATTGATGG	ATCCAGATATCACCGCTATCCGG
ybeJ validate[b]	TGTAAGCAATGAAATCAGCCGC	AGCGTTAACTGCTACCCTCG
gapA qPCR[c]	CTGCTGAAGGCGAAATGAAAGG	TTCAGAGCGATACCCGCTTTAG
dtpA qPCR[c]	CAGCGTCTGATGGGCTTCATTA	GTTATCCGGCACAGCCATCATA
dtpB qPCR[c]	TGGGCTTTATTCTCGGGATGTG	ACGTCTCAAGTGGATCGGTAATG
dtpC qPCR[c]	GTCTGGCTGAAGTTTGCCTTTG	CGGATATCATCACGCCCATTGA
dtpD qPCR[c]	TGCCCTGCTGATCCTCTATCT	CCGAGGATTGGCGTGACATATAC
oppA qPCR[c]	AGTTACCGATGTCAACCGCTAC	GAACTTCGTCCGGGATCTCTTT
dppA qPCR[c]	GTGCTACCGACGACCACAATAA	CTTTGCGTACCGGTTCAAACAC
dppB qPCR[c]	GCTGGTTGATTGACGCACTG	CCAGCAGGTTGACGAGGATAAT

a = primers for generating Lambda Red mutants

b = primers for verifying Lambda Red mutants

c = primers for qRT-PCR

Supplemental Datasets

Dataset S1 (separate file). *E. coli* genomes used for BLASTP analysis.

Dataset S2 (separate file). RNA-seq from clinical UPEC during human UTI.

Tab **S2A** is strains & conditions, **S2B** all up-regulated genes, **S2C** all down-regulated genes

Dataset S3 (separate file). Raw sequence counts from *in vivo* Tn-seq experiments.

Tab **S3A** is Tn-seq urine values, **S3B** Tn-seq bladder values, **S3C** Tn-seq kidney values

Dataset S4 (separate file). Classification of each Tn mutant tested in screening experiments.

Supplemental References

1. R. D. Olson *et al.*, Introducing the Bacterial and Viral Bioinformatics Resource Center (BV-BRC): a resource combining PATRIC, IRD and ViPR. *Nucleic Acids Res* **51**, D678-D689 (2023).
2. A. R. Wattam *et al.*, Improvements to PATRIC, the all-bacterial Bioinformatics Database and Analysis Resource Center. *Nucleic Acids Res* **45**, D535-D542 (2017).
3. A. E. Frick-Cheng *et al.*, Ferric Citrate Uptake Is a Virulence Factor in Uropathogenic Escherichia coli. *mBio* **13**, e0103522 (2022).
4. C. A. Broberg, W. Wu, J. D. Cavalcoli, V. L. Miller, M. A. Bachman, Complete Genome Sequence of Klebsiella pneumoniae Strain ATCC 43816 KPPR1, a Rifampin-Resistant Mutant Commonly Used in Animal, Genetic, and Molecular Biology Studies. *Genome Announc* **2** (2014).
5. H. L. Mobley, J. W. Warren, Urease-positive bacteriuria and obstruction of long-term urinary catheters. *J Clin Microbiol* **25**, 2216-2217 (1987).
6. M. T. Anderson, L. A. Mitchell, L. Zhao, H. L. T. Mobley, Capsule Production and Glucose Metabolism Dictate Fitness during. *mBio* **8** (2017).
7. M. T. Anderson, L. A. Mitchell, L. Zhao, H. L. T. Mobley, Citrobacter freundii fitness during bloodstream infection. *Sci Rep* **8**, 11792 (2018).
8. H. L. Mobley, G. R. Chippendale, M. H. Fraiman, J. H. Tenney, J. W. Warren, Variable phenotypes of Providencia stuartii due to plasmid-encoded traits. *J Clin Microbiol* **22**, 851-853 (1985).
9. M. D. Adams *et al.*, Comparative genome sequence analysis of multidrug-resistant Acinetobacter baumannii. *J Bacteriol* **190**, 8053-8064 (2008).
10. S. J. Hultgren, W. R. Schwan, A. J. Schaeffer, J. L. Duncan, Regulation of production of type 1 pili among urinary tract isolates of Escherichia coli. *Infect Immun* **54**, 613-620 (1986).
11. R. A. Welch *et al.*, Extensive mosaic structure revealed by the complete genome sequence of uropathogenic Escherichia coli. *Proc Natl Acad Sci U S A* **99**, 17020-17024 (2002).
12. M. Kanehisa, S. Goto, KEGG: kyoto encyclopedia of genes and genomes. *Nucleic Acids Res* **28**, 27-30 (2000).
13. M. H. Saier *et al.*, The Transporter Classification Database (TCDB): recent advances. *Nucleic Acids Res* **44**, D372-379 (2016).
14. A. L. Goodman, M. Wu, J. I. Gordon, Identifying microbial fitness determinants by insertion sequencing using genome-wide transposon mutant libraries. *Nat Protoc* **6**, 1969-1980 (2011).
15. N. Wang, E. A. Ozer, A Method for Bioinformatic Analysis of Transposon Insertion Sequencing (INSeq) Results for Identification of Microbial Fitness Determinants. *Methods Mol Biol* **1498**, 243-253 (2017).
16. A. Sintsova *et al.*, Genetically diverse uropathogenic Escherichia coli adopt a common transcriptional program in patients with UTIs. *Elife* **8** (2019).
